# Characterization and treatment monitoring of ureagenesis disorders using stable isotopes

**DOI:** 10.1038/s44324-025-00051-8

**Published:** 2025-05-06

**Authors:** Gabriella Allegri, Martin Poms, Nadia Zürcher, Véronique Rüfenacht, Nicole Rimann, Déborah Mathis, Beat Thöny, Matthias Gautschi, Ralf A. Husain, Daniela Karall, Karolina Orchel-Szastak, Francesco Porta, Dominique Roland, Barbara Siri, Carlo Dionisi-Vici, René Santer, Johannes Häberle

**Affiliations:** 1 Division of Metabolism and Children’s Research Centre (CRC), University Children’s Hospital Zurich, Zurich, Switzerland; 2 Division Clinical Chemistry and Biochemistry, University Children’s Hospital Zurich, Zurich, Switzerland; 3 University Institute of Clinical Chemistry, Inselspital, Bern University Hospital, University of Bern, Bern, Switzerland; 4 Division of Paediatric Endocrinology, Diabetology and Metabolism, Department of Paediatrics, and Institute of Clinical Chemistry, Inselspital, Bern University Hospital, University of Bern, Bern, Switzerland; 5 Centre for Inborn Metabolic Disorders, Department of Neuropediatrics, Jena University Hospital, Jena, Germany; 6 Medical University of Innsbruck, Clinic for Paediatrics, Division of Inherited Metabolic Disorders, Innsbruck, Austria; 7 Department of Paediatrics, Rheumatology and Rare Diseases, University Children’s Hospital, Krakow, Poland; 8 Department of Pediatrics, AOU Citta della Salute e della Scienza, University of Torino, Turin, Italy; 9 Centre des Maladies Héréditaires du Métabolisme, Département de Génétique Humaine, Institut de Pathologie et de Génétique, Gosselies, Belgium; 10 Division of Metabolic Diseases and Hepatology, Bambino Gesù Children’s Hospital IRCCS, Rome, Italy; 11 Department of Pediatrics, University Medical Center Eppendorf, Hamburg, Germany

**Keywords:** Biochemistry, Endocrine system and metabolic diseases, Metabolic disorders

## Abstract

Urea cycle disorders (UCDs) are a group of rare conditions, possibly life-threatening and without definitive cure besides liver transplantation. Traditional biochemical analyses/biomarkers cannot reliably determine changes in the UC-function from baseline to post-intervention. We describe a UHPLC-HRMS method to assess ureagenesis in plasma and dried blood spots for [^15^N]urea and [^15^N]amino acids, using [^15^N]ammonium chloride as tracer. [^15^N]enrichment of urea and amino acids was studied in controls (*n* = 22) and patients (*n* = 59), the latter showing characteristic ureagenesis variations according to their underlying metabolic defect. Follow-up of therapies was successful, as we observed restoration of [^15^N]urea production and lowering of [^15^N]glutamine. There were no adverse events, and only minimal amounts of tracer and samples required with a short sample preparation time and analysis. Thus, the method proved to be safe and efficient to monitor UCD patients of variable severity pre- and post-therapy, being suitable as physiological endpoint for development of therapies.

## Introduction

Ureagenesis is the process of urea production through the urea cycle (UC), which is the main pathway in humans for ammonia detoxification^1^. Impairment of ureagenesis can lead to hyperammonemia, a life-threatening condition that can affect patients at any age and may lead to encephalopathy and to irreversible brain damage depending on the extent and duration of the elevated ammonia levels^2–4^. There are in principle two underlying causes: “primary hyperammonemia” due to urea cycle disorders (UCDs), a group of rare inherited metabolic diseases (1:35,000^5^), in which one of the six enzymes or two transporters that are directly involved in UC function are impaired; “secondary hyperammonemia” due to several inherited or acquired conditions with indirect impact on UC function such as rare inherited metabolic disorders, for instance organic acidemias or fatty acid oxidation defects, or, more commonly, acute or chronic liver failure of various origin^6^.

UCDs exhibit highly variable biochemical and clinical traits^7–9^. Although plasma ammonia and amino acids, and urinary orotic acid values are useful for diagnostic purposes of UCDs, they lack reliability to monitor treatment and to test the efficacy of new therapies^10,11^. For instance, although an increased ammonia concentration is a general indicator of the overall UC function, this parameter is not reliable for a short- or long-term assessment, since blood ammonia concentrations largely fluctuate and depend on various circumstances, including the feeding state^12,13^. Since novel therapies are urgently needed for UCD patients and in fact are already in development, precise measurement of the UC function is a prerequisite for testing their efficacy rendering necessary to establish a simple, low-invasive, accurate assay to assess ureagenesis. Novel treatment approaches for UCDs currently in pre-clinical assessment include gene addition and gene correction as well as cellular therapies^14–22^.

As single ammonia determinations are not ideal as endpoint of interventional studies and for monitoring of novel therapies, the use of stable isotopes to quantify ureagenesis as means of the flux through the UC can be a promising alternative. As a main advantage of stable isotopes for use in biochemical and metabolic studies, they exhibit essentially the same characteristics as the endogenous molecule, do not emit radioactivity, and are detectable at lowest concentrations by mass spectrometric techniques^23^. For in vivo ureagenesis studies using a single oral bolus dose, 2 isotopes have been used as tracers: ^13^C and ^15^N, in the form of [1-^13^C]- or -[^13^C_2_]acetate and ^15^NH_4_Cl (ammonium chloride), respectively^1,23–27^. Although the experimental design is the same for these tracers (collection of a pre-ingestion baseline sample and subsequent sample collections at different time points), each isotope is measured by different techniques and has advantages and disadvantages.

**Fig. 1:**
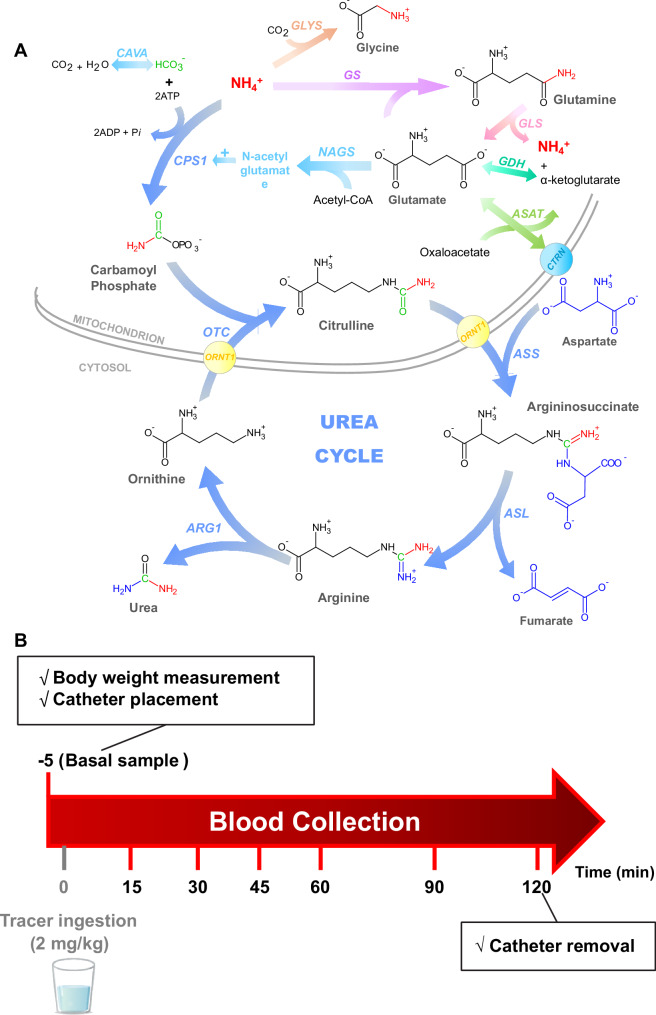
The biochemical background of ureagenesis determination. **A** Fate of ^15^NH_4_^*+*^ tracer after oral ingestion; the exogenous labeled tracer molecule (in red) here shown as “NH_4_^+^” at entry of the urea cycle and as “NH_2_” in intermediate urea cycle metabolites and urea. **B** Design of the ureagenesis assay used in this study. ARG: arginase 1; ASAT: aspartate aminotransferase; ASL: argininosuccinate lyase; ASS argininosuccinate synthetase, CAVA carbonic anhydrase VA, CTRN citrin (mitochondrial aspartate glutamate carrier 2), CPS1 carbamoylphosphate synthetase 1, GDH glutamate dehydrogenase, GLYS glycine synthase (in order to simplify the pathway and focus only in main ammonia metabolism, the glycine cleavage system was omitted here), GS glutamine synthetase, NAGS N-acetylglutamate synthase, ORNT1 mitochondrial ornithine transporter 1, OTC ornithine transcarbamylase.

^13^C tracers are measured by isotope ratio mass spectrometry (IRMS) and assess the UC function indirectly. The main advantage of this tracer is that it’s considered safe since no extra nitrogen is given to patients that are prone to hyperammonemia^26^. The labeled acetate is first rapidly metabolized through the tricarboxylic acid cycle to ^13^CO_2_, subsequently to [^13^C]bicarbonate (H^13^CO_3_^−^) by carbonic anhydrase (variant Va, CAVA) and only then it will be incorporated to [^13^C]carbamoylphosphate, via carbamoylphosphate synthetase 1 (CPS1, EC 6.3.4.16), the first and committing step of the UC (Fig. 1A). As a result, and possibly great drawback of this method, >99% of the tracer is excreted as ^13^CO_2_ in breath, and <1% will enter the UC to produce [^13^C]urea^28^. Although IRMS has a high sensitivity and accuracy in isotopic determinations, it requires that the analytes are isolated, purified and oxidized (combusted) to CO_2_ prior to measurement. This means that [^13^C]urea is not directly analyzed; only after a laborious sample preparation (requiring around 4 h), where [^13^C]urea is finally decomposed to ^13^CO_2_ via urease, the gases (^13^CO_2_/^12^CO_2_) are measured^26,28^. Moreover, IRMS requires relatively large amounts of sample (around 500 µL of plasma for each time point), which can be limiting when considering infant patients. In addition, for accurate interpretation of the measurements in blood, it is necessary to quantify the formation of ^13^CO_2_ in breath^28^.

^15^NH_4_Cl was the first tracer used to assess ureagenesis in UCD patients in 1996^29^. The labeled urea was analyzed by gas chromatography coupled to mass spectrometry (GC-MS). This method allows the rapid separation of analytes through a chromatographic column, before entering in the MS, requiring therefore smaller sample volumes (around 100 µL)^29^. GC-MS is several orders of magnitude less sensitive than IRMS in detecting isotopic abundance. Moreover, it is necessary to transform urea in a volatile and thermostable product using derivatizing agents^29^. The lack of sensitivity of the GC-MS is overcome by the nature of the tracer as >46% of the [^15^N]ammonium ions are directly incorporated in the UC as [^15^N]carbamoylphosphate and subsequently to [^15^N]urea (Fig. 1A)^30^.

With the advent of newer and more sensitive high-resolution mass spectrometers coupled to liquid chromatography (LC-HRMS), we decided for ^15^NH_4_Cl as a tracer, hereby using a very low dose without any risk to UCD patients. In fact, the dose tested in this study is 10 times lower of what was used in 1996 by Yudkoff and colleagues (2 mg/kg, 0.037 mmol/kg versus 20 mg/kg, 0.37 mmol/kg) and represents only around 0.55% of the total daily nitrogen allowance for a severe UCD patient^29,31^. Furthermore, we focused on a fast and simple sample preparation, using a low sample volume (20 µL per time point), which would pose no problem of multiple blood draws even in newborns or infants. In addition, we included the analysis of [^15^N]amino acids to have a broader understanding of subtle changes in the UC and adjacent metabolic pathways and also tested the feasibility of the use of dried blood spots (DBS). We present here the validation results in different matrices (plasma, DBS) and the application of this method in controls and UCD patients, and propose this newly developed ureagenesis assay as reliable, sensitive, and low-invasive and therefore as an excellent tool for evaluating emerging novel UCD therapies.

## Results

### Ureagenesis assay using 2 mg/kg ^15^NH_4_Cl is safe

**Fig. 2:**
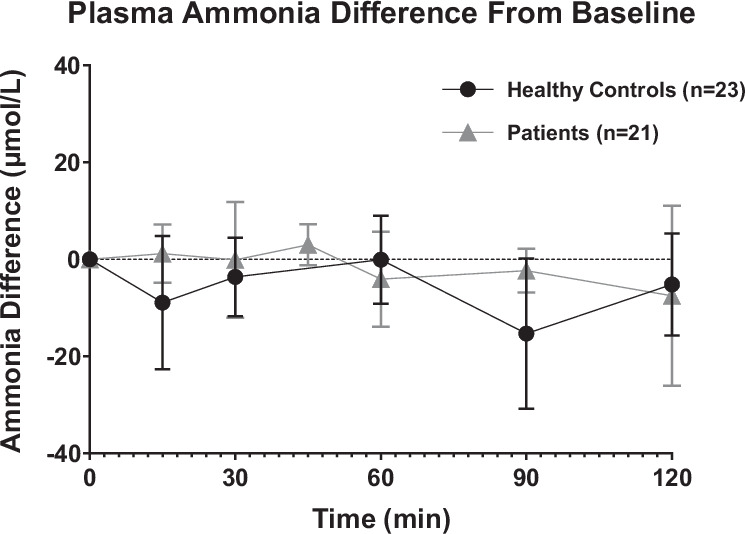
Ammonia difference from basal sample during ureagenesis assay. Results are expressed as mean ± standard deviation for the healthy controls (●) and patients (▲) groups. Ammonia was measured by routine clinical chemistry laboratory at the site where the assay was performed. Since this was a multi-center study, not all time points were measured for ammonia and not all patients had any ammonia measurement.

No adverse events were observed in 39 investigations in 22 healthy subjects and in 74 investigations in any of the 59 patients on the course of the assay. Moreover, the ^15^NH_4_Cl tracer, in the used dose, was considered safe since no significant alterations in ammonia (Fig. 2) between baseline and measurements during the test were observed.

### Tracer recovery

The tracer recovery was calculated as the amount of metabolized tracer observed as the sum of enrichment (areas under the curve) of urea, arginine, citrulline, glutamine, glutamate and glycine divided by the total amount of tracer administered. The tracer recovery in healthy controls after ingestion of 2 mg/kg (~0.037 mmol/kg) of the tracer was 51 ± 10.7%, being in line with what was described by Patterson and colleagues in 1995, where they observed 46% of the tracer in the UC^30^. Patients, depending on the severity of the disease, had a variable tracer recovery (Suppl. Table 2). Body mass index (BMI) seemed to have no influence in tracer absorption in controls and patients and no pattern was observed per disease (Suppl. Fig. 1A, B).

### Ureagenesis analysis of healthy subjects and patients

**Fig. 3:**
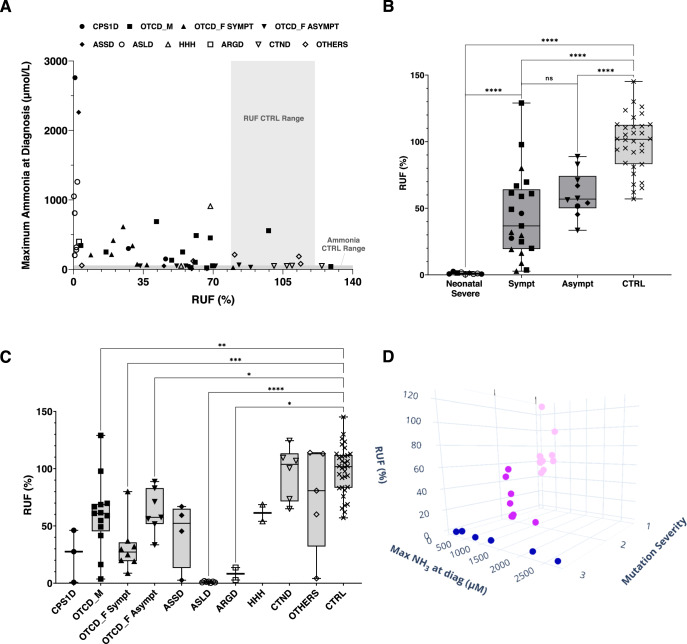
Relative ureagenesis function. **A** Ammonia at diagnosis and RUF (relative ureagenesis function) for all patients; results are shown for individual patients using different geometric symbols for each single disease. Control range for RUF is indicated as a light gray box. Plasma ammonia control range is shown as thickened light gray line at the bottom. **B** RUF from patients according to severity and presence (Sympt) or absence (Asympt) of symptoms. Only patients with an enzymatic defect in the urea cycle were included. Boxes show the median, and the minimum and maximum values for each group; **C** RUF values per disease of all patients. Not significant comparisons were omitted from the graph for clarity purposes; **D** 3D graph of mutation severity, maximum ammonia at diagnosis and RUF. Mutations were classified mild (1 in light pink) intermediate (2 in pink) or severe (3 in blue). This graph only includes patients with an enzymatic UCD defect (*n* = 27). OTC females were excluded due to X-inactivation making the classification of mutation not useful (graph was made using plotly: https://chart-studio.plotly.com). ARGD: arginase 1 deficiency; ASLD: argininosuccinate lyase deficiency; ASSD: argininosuccinate synthetase deficiency; CTND: citrin deficiency; CPS1D: carbamoylphosphate synthetase 1 deficiency; HHH: hyperornithinemia, hyperammonemia and homocitrullinuria. OTCD_M: ornithine transcarbamylase deficiency in males, OTCD_F_SYMPT: OTCD females symptomatic; OTCD_F_ASYMPT: OTCD females asymptomatic. The “Others” group includes: dihydrolipoamide dehydrogenase deficiency, hepatic encephalopathy and chronic liver disease, lysinuric protein intolerance, propionic acidemia, and transmembrane protein 70 deficiency. All the results here obtained were from plasma samples. ****p < 0.0001, ***p < 0.001, *p < 0.05, ns: not significant.

The relative ureagenesis function (RUF) is a measure that quantifies the proportion of an isotopic tracer metabolized through the urea cycle, allowing for comparisons between patients and controls. The reference range of the RUF (79–121%) was calculated as the average RUF ± SD from controls. Intra-individual variation for the RUF was between 3.4–17.0% and was tested in 5 healthy subjects, in which the assay was repeated at least 2 times. It was observed that patients with high plasma ammonia at diagnosis (over 500 µmol/L) would have lower RUF, while ammonia levels up to 500 µmol/L are not automatically correlated with low RUF values (Fig. 3A).

Patients with a severe neonatal onset had lower RUF when compared to symptomatic and asymptomatic patients (Fig. 3B). The symptomatic group was very heterogeneous in terms of RUF (Fig. 3A), especially coming from the ornithine transcarbamylase (OTC) deficient male group. The two symptomatic unrelated OTCD male patients with the highest RUF (Fig. 3B) share the same *OTC* variant (c.264A > T, p.(Lys88Asn)) with two other siblings, where the RUF varies from 49 to 129% and all present with mild symptoms. It is important to note that symptoms were reported as free text from the different centers of this multicenter study without using standardized disease nosology terms, therefore patient group classification may include some subjectivity.

Transporter defects and other related metabolic disorders did not show a RUF significantly different from controls but numbers are small for those subgroups (Fig. 3C). CPS1 deficiency and argininosuccinate synthetase (ASS) deficiency groups were also not significantly different from controls, but again likely due to low number of patients (*n* = 3 and 4, respectively) and the broad range of RUF values (from close to 0 to over 70%). Three of the ASSD patients had the same *ASS1* variant c.535T > C, p.(Trp179Arg) in at least one allele (being one homozygous) - all were mild in their symptoms and their RUF ranged from 45.35 to 67%.

**Fig. 4:**
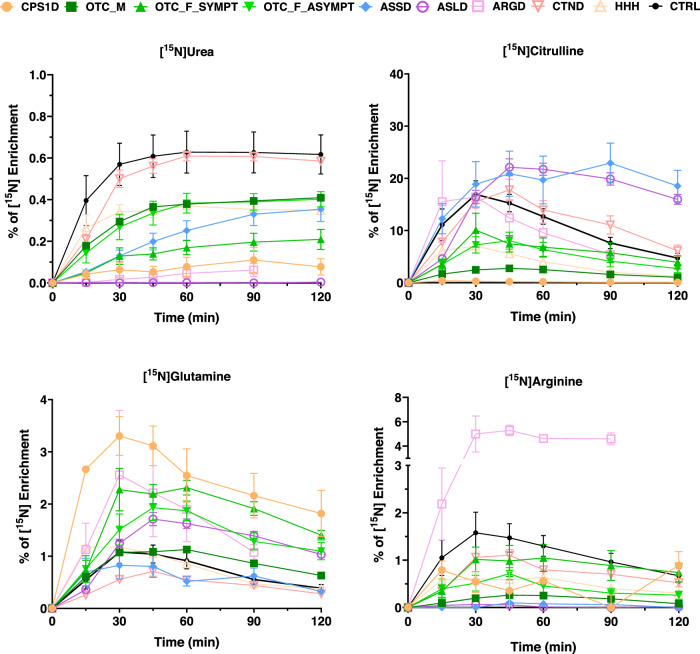
[^15^N] enrichment for urea and selected amino acids for each urea cycle disorder. Curves of %[^15^N] enrichment for urea and selected amino acids for each urea cycle disorder. Control curves (in black) are shown as average ± 3x standard error of the mean. Disease groups are shown as average ± standard error of the mean. ARGD arginase 1 deficiency, ASLD argininosuccinate lyase deficiency, ASSD argininosuccinate synthetase deficiency, CTND citrin deficiency, CPS1D carbamoylphosphate synthetase 1 deficiency, HHH hyperornithinemia, hyperammonemia and homocitrullinuria. OTCD_M ornithine transcarbamylase deficiency in males, OTCD_F_SYMPT OTCD symptomatic females, OTCD_F_ASYMPT OTCD asymptomatic females. All the results here obtained were from plasma samples.

After classifying the patient variants as mild, intermediate or severe according to their impact on the mutated protein, and plotting along with values of RUF and ammonia at diagnosis – hereby including patients with enzymatic defects in the UC but excluding symptomatic and asymptomatic OTCD females because of the unpredictable effect of X-inactivation—we could observe that patients with mild variants had the highest RUF and lowest ammonia while patients carrying severe variants would have the lowest RUF and the highest ammonia at diagnosis (Fig. 3D). The discriminative power of the ureagenesis analysis can be visualized by [^15^N]urea enrichment curves from patients compared to controls, enabling the observation of disease-specific patterns (Fig. 4).

**Fig. 5:**
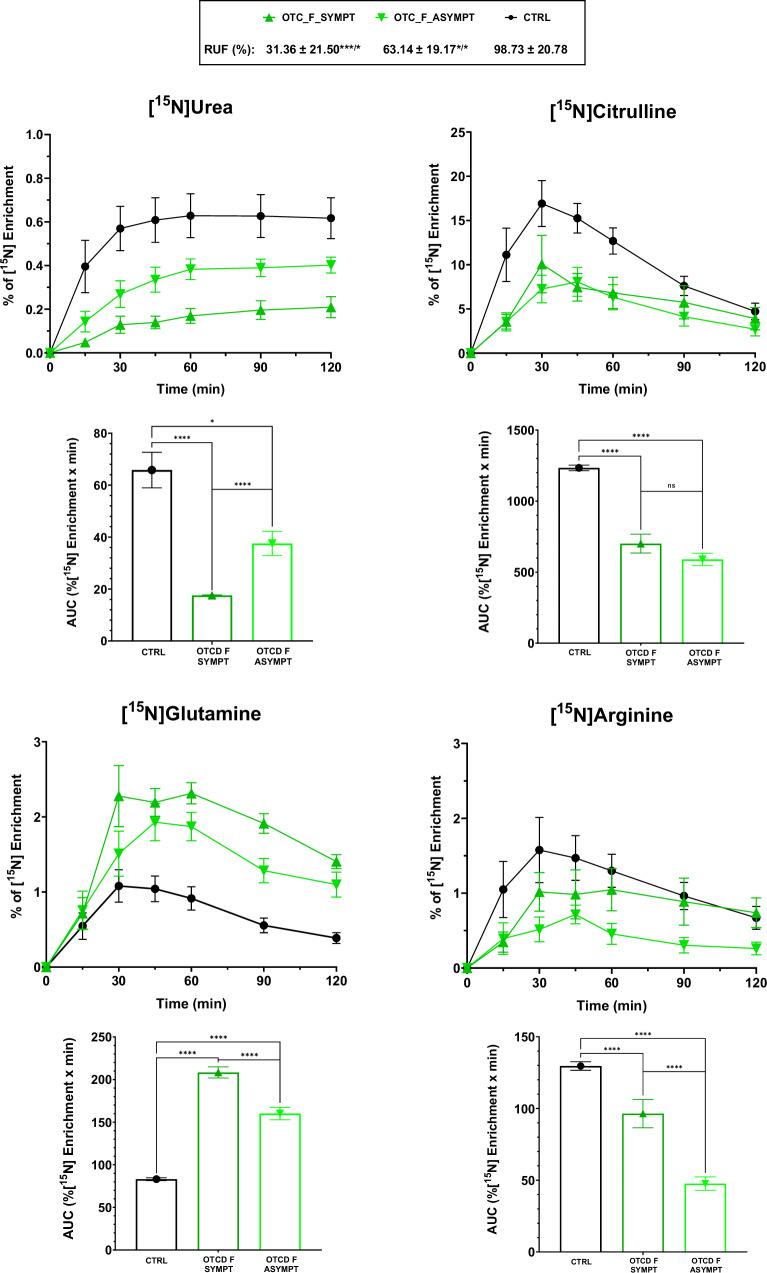
[^15^N] enrichment for urea and selected amino acids enrichment for symptomatic OTCD females, asymptomatic OTCD females and controls. Average ± standard error of the mean of [^15^N]urea and [^15^N]amino acids enrichment in plasma for symptomatic females (OTCD_F_SYMPT, *n* = 10) and asymptomatic females (OTCD_F_ASYMPT, *n* = 8). Control curves (in black) are shown as average ± 3x standard error of the mean. Area Under the Curve (AUC, %[^15^N] enrichment per minute) per group graphs are represented below the respective enrichment curves. In the box above the graphs, RUF values are displayed per group; superscript symbols are a comparison to controls and the “*” (depicted after "/") represents the comparison between symptomatic and asymptomatic females. ****p < 0.0001, ***p < 0.001, *p < 0.05.

It was possible to differentiate controls from UCD patients with lower dose of the tracer based on the percentage of [^15^N]urea and [^15^N]amino acids enrichment. Male OTC deficient patients in this study cohort were exclusively affected by non-severe *OTC* variants. This reflects the fact that many severe OTC deficient patients die early in life^32,33^, and are therefore not in regular clinical follow-up. Regarding the symptomatic OTCD females, the RUF values were significantly lower from controls and asymptomatic groups (Fig. 5). Observing the %[^15^N] enrichment curves and their respective area under the curve (AUC), it was possible to differentiate symptomatic from asymptomatic females and from controls (Fig. 5). The elevated glutamine in the asymptomatic group is in agreement with studies in this group of patients that had demonstrated that biochemical alterations can be detected in apparently asymptomatic OTCD females (2008, 2010)^34,35^.

An interesting observation that needs further investigation was the very low [^15^N]glycine enrichment in all citrin deficient patients (<10% of the control group, Suppl. Fig. 2). It’s important to notice that the respective patients were not taking any sodium benzoate that could otherwise explain the low plasma glycine. A possible explanation of the decrease in glycine concentration is the known reduction of mitochondrial NADH in citrin deficiency hampering the formation of glycine through glycine synthase, which refers to the glycine cleavage system when operating in the reverse direction.

From all the patients analyzed, only four had enzyme activity measured as part of their diagnostic work-up. Patients ASSD_1 (no ASS activity detected) and OTCD_14 (41% OTC activity of controls) had their enzyme activity measured in liver biopsies while patients OTCD_22 and OTCD_23 had their OTC activity measured in plasma (68% and 37% of the median of female controls, respectively)^36^. Interestingly, the RUF for these patients were in agreement with the aforementioned enzyme studies (ASSD_1: 2.62%, OTCD_14: 41.4%, OTCD_22: 51.7 and OTCD_23: 57.5%, respectively).

### Monitoring the efficacy of treatments in UCD patients

**Fig. 6:**
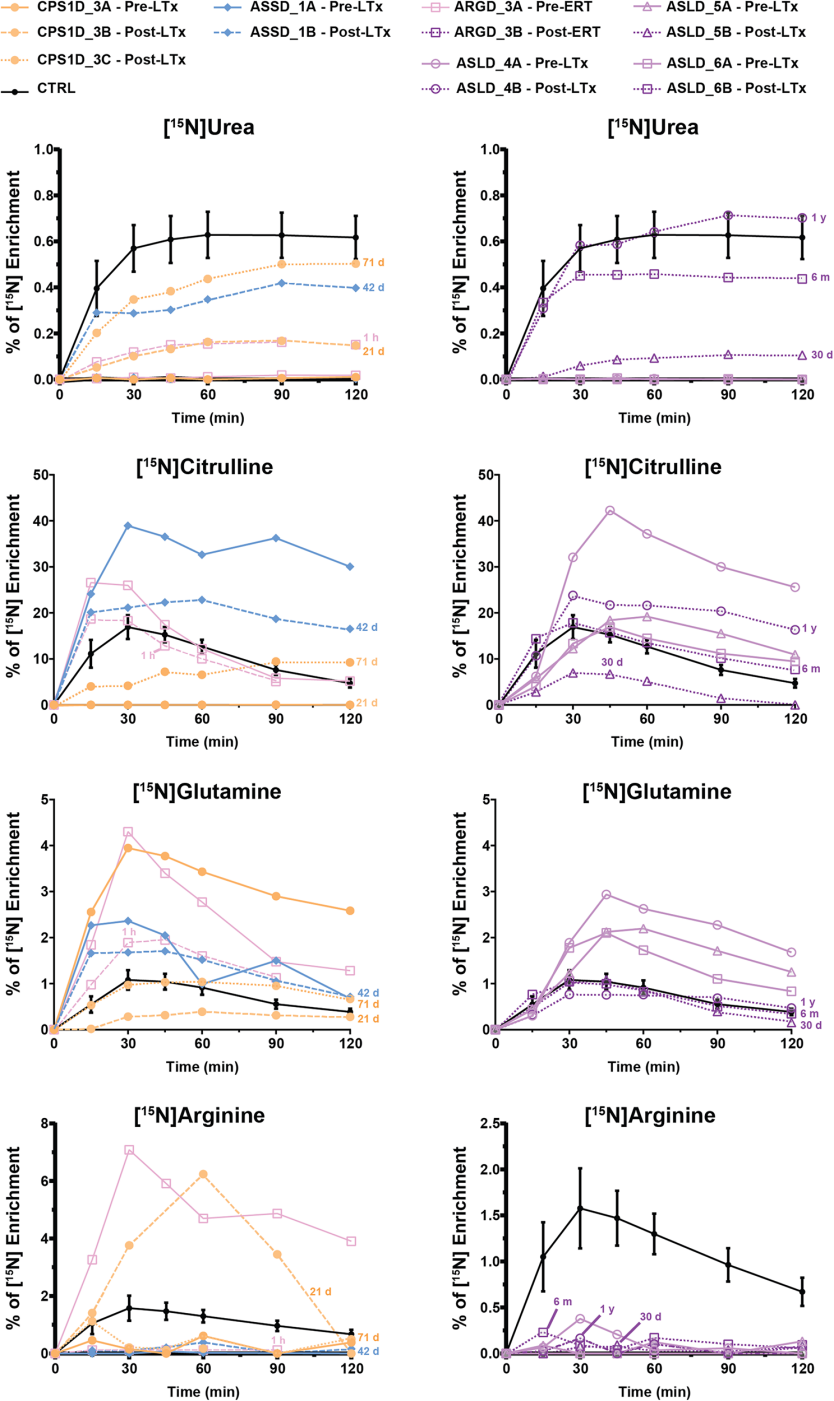
[^15^N] enrichment of urea and selected amino acids of patients pre- and post-therapy. Curves of %[^15^N] enrichment of urea and selected amino acids in plasma of patients pre- and post-therapy. Full lines represent pre-therapy and dashed lines post-therapy results. Time post-therapy is showed in the graph as hours (h), days (d), months (m) or years (y) for each patient. ARGD arginase 1 deficiency, ASLD argininosuccinate lyase deficiency, ASSD argininosuccinate synthetase deficiency, CPS1D carbamoylphosphate synthetase 1 deficiency, ERT enzyme replacement therapy, LTx liver transplantation. Control (CTRL) curves are shown as average ± 3x standard error of the mean for [^15^N]urea and [^15^N]amino acids enrichment.

**Fig. 7:**
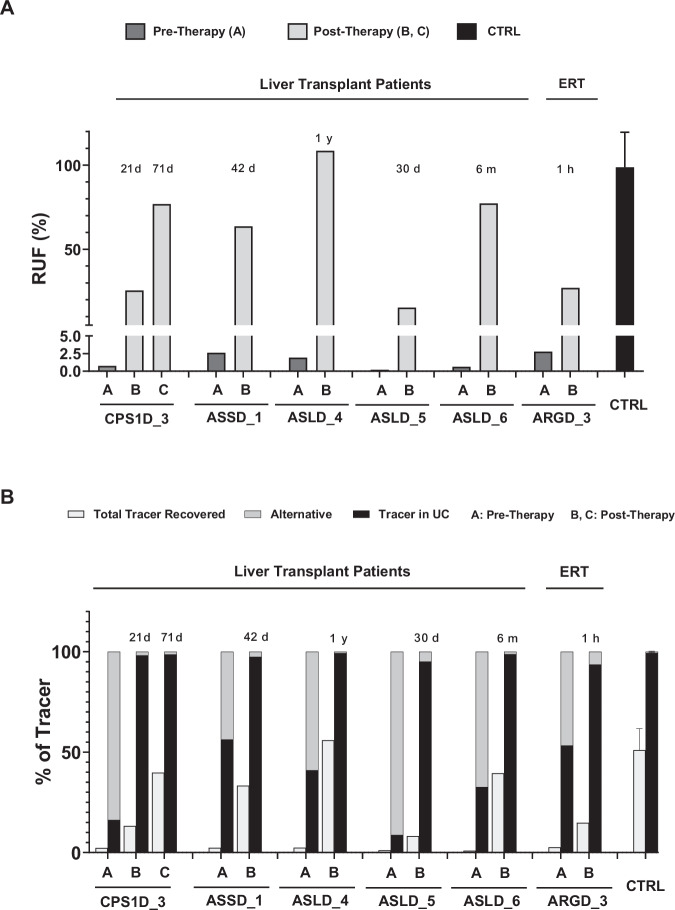
Follow-up of therapies in patients showing. **A** RUF (%) pre- and post-therapy and (**B**). total tracer recovered and % of the tracer in the urea cycle and in alternative pathways in patients pre- and post-therapy. The time post-therapy is described on top of the bar. ERT: enzyme replacement therapy. Time post-therapy is shown in the graph as hours (h), days (d), months (m) or years (y) for each patient. Control (CTRL) values are shown as average ± standard deviation. All the results here obtained were from plasma samples. ARGD arginase 1 deficiency, ASLD argininosuccinate lyase deficiency, ASSD argininosuccinate synthetase deficiency, CPS1D carbamoylphosphate synthetase 1 deficiency.

In our patient cohort, liver transplantation was performed in six patients affected by different disorders, who all underwent ureagenesis analysis before the procedure and thereafter at different time points. In five patients, we could confirm the restoration of UC function (Figs. 6 and 7). In one patient, the post-therapy samples could not be analyzed due to unsatisfactory pre-analytical sample processing. One patient underwent enzyme replacement therapy (ERT) for arginase deficiency and also showed improvement of the RUF values despite the fact that the ERT was mainly targeting plasma arginine cleavage and not hepatic arginase restoration^37^; this finding may indicate that the ornithine liberated by plasma arginase action provides substrate to the hepatic OTC reaction, an observation expanding our understanding of nitrogen metabolism.

As a general outlook of therapy efficacy monitoring in UCDs, enrichments of [^15^N]urea should be studied comprehensively together with [^15^N]glutamine and disease-relevant labeled amino acids, as it is possible to observe the urea production increase and the change in other amino acids enrichment post-therapy in all patients (Fig. 6). Studies of enrichment of citrulline and arginine were useful depending on the metabolic defect; for instance, in the arginase deficiency case, it was possible to observe the reduction in the enriched arginine already after 1h of enzyme replacement therapy. Moreover, in ASS and argininosuccinate lyase (ASL) deficient patients, it was possible to observe the decrease in [^15^N]citrulline enrichment post-therapy (Fig. 6). Likewise, it is possible to observe the increase in RUF (Fig. 7A) and the increase in tracer recovery post-therapy (Fig. 7B). Finally, as an interesting outlook of the ureagenesis assay here studied, it was possible to follow-up the post-therapy efficacy by the increase of the ^15^N label being metabolized through the UC and the decreased diversion of the tracer into alternative pathways (such as glutamine production) (Fig. 7B).

## Discussion

UCDs are rare or even ultra-rare inherited metabolic conditions currently treated with dietary interventions (mainly low-protein diet plus supplementation of essential amino acids) and/or drugs (mainly nitrogen scavengers) for enhancing the excretion of potentially neurotoxic nitrogenous compounds^31^. Despite improvements in the outcome of patients, both regarding survival and quality of life, neonatal onset patients or those suffering severe metabolic crises later in life have an overall poor prognosis^32,33,38–40^. Therefore, there is a clear urgent need for novel therapies. Fortunately, due to joint and parallel efforts from academia and industry, there are several novel therapies planned, including for example, messenger RNA (mRNA) therapy (ClinicalTrials.gov Identifiers: NCT05526066 and NCT06488313) and recombinant adeno-associated virus (AAV) therapy (ClinicalTrials.gov Identifier: NCT05345171, NCT02991144 and NCT05092685) for OTC deficiency. As expected from recent pre-clinical publications describing the use of novel CRISPR Cas9-based gene editing^41–43^, clinical trials applying these approaches are also emerging for UCDs (ClinicalTrials.gov Identifier: NCT06255782). However, accurate measurement of the efficacy of these interventions remains challenging. Traditional biochemical analyses and biomarkers cannot reliably determine the change in UC function from baseline to post-intervention, which would be highly attractive and could even serve as a clinically meaningful endpoint. To achieve this, we aimed at developing a fast, sensitive, and low-invasive stable isotope-based assay, which directly measures UC function in vivo and can be used for diagnostics and monitoring, and for clinical studies such as novel therapy development.

The method here presented to evaluate in vivo ureagenesis exhibits several advantages when compared with previous ones; it uses the separation capacity through chromatography, which requires lower sample volume, and HRMS, which increases sensitivity. The small sample volume becomes extremely relevant when performing the assay in infants and gives the possibility of the repetition of the assay after a short time period. Another advantage is the fast sample preparation time; from one sample, after a simple plasma precipitation with solvent, the same supernatant is used for a direct analysis of urea, without requiring derivatization, and for amino acids analysis after derivatization with 6-Aminoquinolyl-N-hydroxysccinimidyl carbamate (AQC) to increase detection capacity. The overall sample preparation time is <30 min and total run time around 15 min all in a 96 well-plate, which allows a high sample throughput. Moreover, the possibility of using DBS facilitates collaboration with distant centers from any part of the world. If studies aim at monitoring individual patients pre- and post-intervention or for general follow-up, we recommend the use of the same matrix, since, despite a good correlation between plasma and DBS, the assessment would be more precise.

The rationale for the [^15^N]ammonium chloride tracer selection, instead of [1-^13^C] or [^13^C_2_]acetate, was that the high incorporation of the ^15^N label into the UC (~51% in our study) associated with the use of an extremely sensitive mass spectrometry technique would allow a substantial tracer dose reduction. Indeed, it was possible to reduce the dose 10 times of what was used before^29^, also requiring less sample, with no adverse effects. It’s worth to mention that the amount of tracer used represents approximately to 0.55% of the protein per kg that UCD patients are allowed to consume per day^31,44^. In addition, this dose reduction considerably attenuated the unpleasant taste of the tracer and very few complaints about taste and even no gagging were observed, which is different from described before^29^. Moreover, ammonium chloride is a substance used in several common therapeutic products such as expectorant formulas, diuretics, as a food acidifier and as food additive. Another concern towards the use of [^15^N]ammonium chloride, raised by Tuchman and colleagues^28^, is the possible dilution of the tracer in the already large pool of nitrogen of UCD patients, potentially confounding comparisons with control data. However, this issue was mitigated by correcting for the endogenous levels of the specific metabolites analyzed, rather than the total nitrogen.

There was also a concern that the use of nitrogen scavengers would reduce the availability of the ^15^N label and therefore show a spurious low ureagenesis function. Usually, nitrogen scavengers are given in 3 to 4 doses throughout the day. Yudkoff and colleagues in 1996, found no influence in ureagenesis of a 250 mg/kg/day dose of phenylbutyrate (PB) 30 min prior the assay, although it was not discarded that higher doses could have an effect. Therefore, it was suggested that a high PB dose before the assay should be suppressed^29^. Lee and colleagues also assessed the effect of nitrogen scavengers (sodium phenylacetate/benzoate) although performing an alternative and complex assay infusing intravenously [5-^15^N]glutamine and [^18^O]urea for 8 h^24^. In this case, they observed a decrease in ureagenesis, but this was likely due to the capacity of the tracer to react directly with phenylacetate, and form labeled phenylacetylglutamine. As these nitrogen scavengers affect isotopically labeled as well as unlabeled metabolites similarly, the isotopic enrichment of the measured metabolites should however not be affected.

We aimed to provide a fast, sensitive, and low-invasive analysis of the UC function in vivo to be used for diagnostics and monitoring, and for clinical studies such as novel therapy development. This latter point seems to be of superior importance for UCD patients, since pure clinical endpoints such as “number of metabolic crises” are dependent on various factors and thus challenging to validate and use as study endpoints. Likewise, levels of classical UCD biomarkers (plasma ammonia and amino acids) are fluctuating and dependent on various factors including diet, fasting times or activity levels. All these concerns are dispensable with the here proposed objective and comprehensive quantification of the urea cycle function and adjacent metabolic pathways. The ureagenesis analysis will be useful for determination of the efficacy of novel interventions such as gene addition or editing approaches. In the past, these studies mainly relied on a combination of partly unspecific clinical and biochemical endpoints, or required invasive sample collection, for instance liver biopsies. Moreover, the method seems very well-suited for monitoring and to detect a possible drop in therapy success, for instance in case of liver growth and the resulting dilution of gene addition through hepatic cell division. We therefore propose this method as primary endpoint for many of the upcoming studies into mRNA therapy, recombinant adeno-associated virus (AAV) therapy, or CRISPR Cas9-based gene editing.

The functional test proposed here is of particular interest for the evaluation of OTCD females, which are a notoriously difficult to diagnose patient subgroup^45,46^. Enzyme analysis for OTC deficiency usually requires obtaining a liver biopsy sample^31^. In most cases, this is done by transcutaneous ultrasound-guided needle biopsy and often 2 or 3 biopsies are collected from different parts of the liver. It is well documented in female patients that this procedure results in quite variable levels of residual OTC enzyme measurements. This has even been systematically studied in single patients in whom high variability between 5% and 25% OTC activity was found in the same individual^47^. More recently, use of OTC enzyme measurement by determination of the reverse reaction in plasma samples has been proposed as an alternative for the highly invasive liver biopsy^36^. However, there is no report on the correlation between the OTC liver and plasma activity. While the OTC assay in plasma is very reliable in males, the assay cannot distinguish between symptomatic and asymptomatic OTC females. The alternative to enzyme studies would be genetic analysis of the *OTC* gene, which has a known success rate of only 80%^48^, and additional tests can reduce but not entirely close this diagnostic gap^49,50^. Hence, functional testing with the here proposed ureagenesis analysis could be an attractive alternative, and it is important to note that our test can distinguish symptomatic from asymptomatic OTCD females and therefore offers advantages if compared with both liver and plasma OTC enzyme analysis. Unfortunately, with two different methods (liver and plasma OTC activity) and the low the number of patients (*n* = 4) who underwent enzymatic testing, we were not able to establish a proper correlation between the respective RUF values and enzyme activities.

An aspect that should be noticed is the extremely low RUF for the 2 youngest patients at the time of the assay, both at neonatal age (OTCD_23, 28 days and HHH_1A,B 7 and 9 days). When the assay was repeated, there was a significant increase in the RUF values in both patients (Suppl. Table 2). We cannot discard the possibility that the lower values in RUF could be due to immaturity of the UC. It is known that in mice, glutamine synthetase is overexpressed in liver at neonatal age, while some UC enzymes are lowly expressed^11^. Our control group unfortunately did not include such young subjects and data from age-matched neonatal human controls will likely always be inaccessible. Therefore, at least caution with result interpretation in patients in the first few weeks should be taken.

Another area of required future studies is the possible impact of the fasting time on the ureagenesis function. Fasting times are possibly relevant either because they may directly impact on the enteral passage and uptake of the tracer, and/or due to the possible stimulation of the urea cycle caused by dietary protein content^51^. In this study, we requested a minimum fasting of 4 h (2 h in patients <2 years of age) as this is the clinical practice for meaningful amino acid determination, however, patient fasting times varied when compared to controls (11.6 ± 1.6 h in controls and 6.9 ± 4.8 h in patients older than 2 years) so no inferences can be made on the impact of this parameter on ureagenesis function. The main reason for the larger variability of the total fasting times were the different test situations of the patients, such as outpatient clinics, day-care clinics, inpatients on normal ward, inpatients on the intensive care unit, while control subjects uniformly underwent the test in the early morning after an overnight fast.

In summary, the stable isotope-based quantification of UC function proved to be a simple, fast and efficient tool for ureagenesis evaluation, hereby serving as a future “biochemical benchmark” that is more meaningful than current biochemical parameters, usually plasma ammonia and amino acids, in patients with impaired ureagenesis. This new test will specifically provide a basis for monitoring the effect of current therapies as well as those hopefully soon being tested in clinical trials.

## Methods

### Material

[^15^N]ammonium chloride ( ≥ 99% isotope enrichment), [^15^N_2_]urea ( ≥ 98% isotope enrichment) and [^15^N_2_, ^18^O, ^13^C]urea ( ≥ 98% isotope enrichment, used as internal standard) were obtained from Cambridge Isotopes (Tewksbury, MA, USA). AccQ-Tag Ultra Derivatization Kit for amino acids analysis was purchased from Waters (Baden, AG, Switzerland).

Urea (certified reference material, 99.4 ± 0.1%), methanol (analytical grade), acetonitrile (ACN, analytical grade), and water containing 0.1% of formic acid were purchased from Sigma-Aldrich (Buchs, SG, Switzerland). Ultrapure water was used for analysis with a conductivity of 18.2 mΩ x cm at 25 °C.

### Subjects

This study was registered at ClinicalTrials.gov (Identifier: NCT05671666). Patients were from different centers across the world. The control group consisted of healthy subjects living in Switzerland (13 females and 9 males, ranging from 19 to 58 years). Sex was not considered as a biological variable. Genotype severity was classified into 3 categories: “mild” for genetic variants on at least one allele known to allow some relevant residual function of the respective gene product; “severe” for genetic variants on both alleles known to result in complete loss of gene product function; “intermediate” for all genotypes not qualifying for category “mild” or “severe”. A concise description of the main characteristics of patients is provided in Table 1 (more complete patient data is described in Suppl. Table 1).

**Table 1:** Main characteristics of patients

Patient ID	Gender	Neonatal onset yes: 1; no: 0	Asympt: 0 Sympt: 1	Affected gene	Variant (s)	Age at assay (y)	NH_3_ at diagnosis (µmol/L)	Additional Information
**CPS1D_1**	F	1	1	***CPS1*** (NM_001875.5)	c.2291C>T, p.(Pro764Leu); c.3935dup, p.(Met1312IlefsTer11)	0.34	289	Low protein diet (1.4 g/kg/d)
**CPS1D_2**	F	0	0		c.4002G>A, p.(Glu1334 = ) splicing; c.4229G>A, p.(Trp1410Ter)	31.8	117	
**CPS1D_3A, B, C**	M	1	1		c.3520C>T, p.(Arg1174Ter); c.4229G>A, p.(Trp1410Ter)	0.95, 1.01, 1.20	2760	A: pre-LTX, B: 21 and C: 71 d post-LTx
**OTCD_1**	M	0	1	***OTC*** (NM_000531.6)	c.604C>T, p.(His202Tyr)	10.7	344	
**OTCD_2**	M	0	1		c.394T>C, p.(Ser132Pro)	13.6	487	
**OTCD_3**	M	0	1		c.264A>T, p.(Lys88Asn)	17.9	557	
**OTCD_4**	M	0	1		c.622G>A, p.(Ala208Thr)	74.4	22	Low protein diet (42 g/d)
**OTCD_5**^**^^**^	M	0	1		c.622G>A, p.(Ala208Thr)	82.5	26	Low protein diet (35 g/d)
**OTCD_6**	M	0	1		c.264A>T, p.(Lys88Asn)	3.9	42	Protein in diet: 26 g/d, 1.6 g/kg/d
**OTCD_7**	M	0	1		deep intronic c.540 + 265G > A	2.1	52.2	positive NBS for low citrulline (3.2 cut off 7.1)
OTCD_8*	M	0	1		c.264A>T, p.(Lys88Asn)	11.3	133	Protein in diet: 38 g/d, 0.87 g/kg/d
OTCD_9*	M	0	1		c.264A>T, p.(Lys88Asn)	14.0	102	Protein in diet: 43 g/d, 0.6 g/kg/d
**OTCD_10**	M	0	1		c.365A>G, p.(Glu122Gly)	2.3		
**OTCD_11**	M	1	1		c.594C>A, p.(Asn198Lys)	0.77		
**OTCD_12**	M	0	1		c.394T>C, p.(Ser132Pro)	34.7	453	
**OTCD_13**	M	0	1		c.216 + 1G > A, splicing	2.8	449	
**OTCD_14**	M	1	1		c.386G>A, p.(Arg129His)	16.6	688	Liver OTC activity showing 40% of WT, neonatal onset on day 6
**OTCD_15**	F	0	1		c.958C>T, p.(Arg320Ter)	6.9	417	
**OTCD_16**^**-**^	F	0	1		c.433C>T, p.(Gln145Ter)	35.9	64	
**OTCD_17**	F	0	1		c.2T>C; p.?	4.0	614	
**OTCD_18**^**-**^	F	0	1		c.718-1G > A, splicing	5.3	340	
**OTCD_19_A, B, C**	F	0	1		c.274C>T, p.(Arg92Ter)	8.5, 8.6, 8.6	208	
**OTCD_20**	F	0	1		not identified	17.7	214	
**OTCD_21**^**^^**^	F	0	1		c.622G>A, p.(Ala208Thr)	53.0	26	Low protein diet (45 g/d)
**OTCD_22**^**#**^	F	0	0		c.698C>T, p.(Ala233Val)	1.3	never HA	Plasma OTC activity (68% of the median of female controls)
**OTCD_23_A**^**#**^**,B**	F	0	0		c.698C>T, p.(Ala233Val)	0.08, 1.4	never HA	Plasma OTC activity and 37% of the median of female controls
**OTCD_24**^**-**^	F	0	0		c.433C>T, p.(Gln145Ter)	2.3		
**OTCD_25**^**-**^	F	0	0		c.718-1G > A, splicing	33.8	never HA	
**OTCD_26_A, B**	F	0	0		c.674C>T, p.(Pro225Leu)	34.2, 35.1	51	mother of OTC male with fatal neonatal onset
**OTCD_27**	F	0	0		c.386G>A, p.(Arg129His)	35.08	never HA	
OTCD_28^*^	F	0	0		c.674C>T, p.(Pro225Leu)			
OTCD_29^*^	F	0	0		c.674C>T, p.(Pro225Leu)			
**ASSD_1A, B**	M	1	1	***ASS1*** (NM_054012.4)	c.1168G>A, p.(Gly390Arg) homozygous	0.21, 0.38	2262	A: pre-LTX, B: 42 d post LTx
**ASSD_2**	M	0	0		c.535T>C, p.(Trp179Arg); c.917T>G, p.(Val306Gly)	8.6	8.8	
**ASSD_3**	M	0	0		c.535T>C, p.(Trp179Arg) homozygous	4.4	8.4	Protein in diet: 25 g/d, 1.3 g/kg/d
**ASSD_4**	M	0	0		c.535T>C, p.(Trp179Arg); c.827T>A, p.(Met276Lys)	4.8	never HA	
**ASLD_1**	F	1	1	***ASL*** (NM_000048.4)	c.470G>T, p.(Gly157Val); r.568_602del, p.(Val190TrpfsTer33)	0.16	281	
**ASLD_2**	F	1	1		c.707G>A, p.(Arg236Gln) homozygous	5.9	317	
**ASLD_3A, B**	F	1	1		c.719-1G > A; splicing homozygous	0.48, 0.81	810	A: pre-LTX, B: 100 d post-LTx
**ASLD_4A, B**	F	1	1		c.436C>T, p.(Arg146Trp) homozygous	7.4, 8.6	1261	A: pre-LTX, B: 1 year post-LTx
**ASLD_5A, B**	M	1	1		c.479A>C, p.(His160Pro) homozygous	0.87, 1.1	1052	A: pre-LTX, B: 30 d post-LTx
**ASLD_6_A, B**	M	1	1		c.1128C>A, p.(Tyr376Ter) homozygous	0.25, 1. 6	204	A: pre-LTX, B: 6 m post-LTx
**ASLD_7A, B**	M	1	1		c.1366C>G, p.(Arg456Gly) homozygous	17.6, 18.1	na	
**ARGD_1**	M	0	1	***ARG1*** (NM_000045.4)	c.306-506A > G, r.305_306ins115, p.(Leu103LysfsTer6)	2.8	110	
**ARGD_2**	M	0	1		not known	7.8	not known	
**ARGD_3A, B**	M	1	1		c.647ins32bp, p.?; c.871C>T, p.(Arg291Ter)	13.9, 14.4	>400	Patient on ERT
**CTND_1**	F	1	1	***SLC25A13*** (NM_014251.3)	c.1628dup, p.(Ile544TyrfsTer24) homozygous	2.5	not elevated	
**CTND_2**^**+**^	F	0	1		c.1311 + 1G > A, splicing; c.1348del, p.(Glu450LysfsTer58)	11.5	not elevated	
**CTND_3**^**+**^	M	0	1		c.1311 + 1G > A, splicing; c.1348del, p.(Glu450LysfsTer58)	17.4	not elevated	
**CTND_4**	M	1	1		c.173_174del, p.(Val58GlyfsTer24); c.1813C>T, p.(Arg605Ter)	4.9	not elevated	
**CTND_5**	M	1	1		c.74C>A, p.(Ala25Glu); c.1078C>T, p.(Arg360Ter)	9.5	not elevated	
**CTND_6**	M	0	1		c.1177 + 1G > A, p.(Ala340_Arg392del); c.1763G>A, p.(Arg588Gln)	52.8	not known	
**HHH_1A, B, C**	M	1	1	***SLC25A15*** (NM_014252.4)	c.535C>T, p.(Arg179Ter) homozygous	0.02, 0.02, 1.8	910	
**HHH_2**	M	0	0		c.380C>T, p.(Thr127 Met) homozygous	9.0	not elevated	
**PA**	M	1	1	*PCCA/B*	-	22.3	slightly elevated	
**HE**	M	0	1	-	-	68.2	186	chronic liver disease, adult, secondary HA, single event, hepatic encephalopathy
**LPI**	M	0	1	***SLC7A7*** (NM_003982.4)	c.726G>A, (p.Trp242Ter) homozygous	9.6	57	
**TMEM70D**	M	0	1	***TMEM70*** (NM_017866.6)	c.317-2A > G, splicing homozygous	8.3	120	
**DLDD**	M	0	1	***DLD*** (NM_000108.5)	c.685G >T, p.(Gly229Cys) homozygous	18.5	211	

*ARGD* arginase 1 deficiency, *ASLD* argininosuccinate lyase deficiency, *ASSD* argininosuccinate synthetase deficiency, *CTND* citrin deficiency, *CPS1D* carbamoylphosphate synthetase 1 deficiency, *DLDD* dihydrolipoamide dehydrogenase deficiency, *ERT* enzyme replacement therapy, *HA* hyperammonemia, *HE* hepatic encephalopathy and chronic liver disease, *HHH* hyperornithinemia, hyperammonemia and homocitrullinuria syndrome, *LPI* lysinuric protein intolerance, *LTx* liver transplantation, *NBS* newborn screening, *OTCD* ornithine transcarbamylase deficiency, *PA* propionic acidemia, *TMEM70D* transmembrane protein 70 deficiency, *na* not available, ^^: father/daughter; +: brother/sister; * mother/ daughter; ** brothers; - mother/ daughter; # aunt/niece.

More detailed information on patients is provided in Suppl. Table 1.

### Study approval

This study was approved by Swissethics (BASEC-No. 2019-01352). All procedures were in accordance with the Declaration of Helsinki of 1975, as revised in 2000. Written informed consent from all patients and/or legally authorized representatives was obtained prior inclusion in the study.

### Ureagenesis assay design in humans

The study was performed as follows (Fig. 1B): prior to the procedure, there was for healthy subjects and patients an age-dependent fasting time of at least 4 h if ≥2 years and of at least 2 h if <2 years. To start the test, an indwelling venous catheter was placed and a blood basal sample was collected in lithium-heparin tube. Afterwards, controls or patients received an oral dose of 2 mg/kg (0.037 mmol/kg) of ^15^NH_4_Cl diluted in water. In the cases where oral administration was not possible, for instance in neonatal patients on the intensive care unit, the solution was given via a nasogastric tube, or could have been given via percutaneous endoscopic gastrostomy tube if already in place. Each 0.5 mL blood samples were collected at 15, 30, 45, 60, 90, and 120 min after tracer administration. Each blood sample was immediately spotted on a filter paper card and allowed to dry at room temperature for around 4 h. Plasma samples were obtained by centrifugation at 2000 × g for 5 min at room temperature.

Concomitant medication as required by the patient was allowed during the assay. The only exception would have been carglumic acid, an allosteric activator of the first UC enzyme CPS1, which would have been stopped for 24 h prior to the test but this drug was not taken by any of the patients included in this study.

### Plasma sample preparation for UHPLC-HRMS analysis

Twenty µL of plasma was mixed with 10 µL of internal standard mix (1.0 mM [^15^N_2_, ^18^O, ^13^C]urea, 40 µM [d5]phenylalanine in methanol) and 70 µL cooled methanol, and was subsequently vortexed and centrifuged (15,000 × g) for 10 min at 4 °C. For urea enrichment analysis, a 10 µL aliquot of supernatant was added to a vial with insert (or 96-well plate well) containing 190 µL of 96% ACN/ 0.1% formic acid. For the amino acid analysis, a 20 µL aliquot of the same supernatant was evaporated, resuspended in 40 µL of borate buffer and vortexed. 10 µL of the AQC reagent in acetonitrile was added the reaction mixture was vortexed and placed on a 55 °C heating block for 10 min (according to Waters protocol). Ten µL of this mixture were added to 190 µL 4% ACN/ 0.1% formic acid. The rest of the supernatant was stored at −80 °C.

### Dried blood spot sample preparation for UHPLC-HRMS analysis

One 3 mm diameter punch was placed in a tube containing 45 µL of water and 35 µL of IS mix. After vortexing, samples were sonicated for 10 min in a water bath. Subsequently, 270 µL of methanol were added to the samples, followed by vortexing and cooling at 4 °C for 10 min. Samples were centrifuged (15,000 × g) for 10 min at 4 °C. For urea enrichment analysis, a 40 µL aliquot was added to a vial with insert containing 160 µL of 96% ACN/ 0.1% formic acid. For the amino acid analysis 180 µL of the same supernatant was added to a vial and evaporated under N_2_ stream at 55 °C. 40 µL of borate buffer, was added and briefly vortexed and then 10 µL of the AQC reagent was added and samples were once more vortexed. Samples were allowed to react at 55 °C for 10 min and 20 µL of the reaction mix was added to 180 µL of 4% ACN/ 0.1% formic acid.

### Non- and labeled-urea and amino acids measurement by UHPLC-HRMS

Analyses were performed using a liquid chromatograph (LC; Dionex UltiMate 3000, Thermo Fisher, Switzerland) coupled to a high resolution mass spectrometer with electron spray ionization source (Q Exactive hybrid quadrupole-Orbitrap, Thermo Fisher, Switzerland) using Acquity BEH Amide column (100 mm x 2.1 mm ID, 1.7 µm dp, Waters, USA) for urea analysis and an Acquity HSS T3 Amide column (150 mm x 2.1 mm ID, 1.8 µm dp, Waters, USA) for amino acid analysis. The mobile phase A was water 0.1% formic acid and the mobile phase B was 95% ACN/water 0.1% formic acid. The gradient program for urea analysis was: start at 80% B until 100% B at 1.4 min, then hold until 2.39 min with a flow of 200 µL/min at 2.4 min 100% A and flow was increased to 400 µL/min and hold until 5.0 min; at 5.1 min the flow was reduced back to 200 µL/min and 80% B until 7.0 min. Column compartment temperature was kept stable at 30 °C and injection volume was 2 µL. For the amino acid analysis the gradient was: with a flow of 400 µL/min, start at 4% B until 0.5 min then increase to 10% B until 2.5 min, then to 28% B until 5.0 min; at 5.1 min increase to 95% and hold for 1 min and at 6.2 return to 4% and B and hold until 7.5 min. Column compartment temperature was 30 °C and injection volume was 10 µL. The ESI-HRMS data were recorded in the positive ion mode with capillary voltage of 3.47 kV, with capillary and auxiliary gas heater temperature of 350 °C. Nitrogen was used as a nebulizing (100 L/h) and a drying (450 L/h) gas. For urea, the full scan range was set to 50.0 –100.1 *m/z* and spectra were recorded in the profile spectrum data at a scan rate of 0.1 s/scan (maximum injection time: 0.1 s) and at a resolution of 70,000 (FWHM). Protonated NL-urea (non-labeled) and [^15^N]urea, [^13^C]urea, [^15^N_2_]urea and [^15^N_2_, ^18^O, ^13^C]urea were identified through comparison of accurate mass with the exact mass (Suppl. Table 12). For amino acids analysis, the full scan range was set from 100.0–500.1 *m/z* and spectra were recorded in profile spectrum mode at a scan rate of 0.1 s/scan (maximum injection time: 0.1 s) and at a resolution of 140,000 (FWHM).

### Data processing

Peak integration was achieved using the Xcalibur™ software package with mass-to-charge and retention time peak detection windows of 0.005 *m/z* and 60 s for urea and 0.007 *m/z* and 100 s the amino acids, respectively.

Data preprocessing included missing value imputation and normalization, where integrated peak areas were scaled according to the respective IS response. To detect aberrant peak integrations, outliers among the triplicate measurements were defined as runs which show a deviation of >30% compared to the other two runs of the respective triplicate and were omitted from further analysis.

### Method validation

While the data on how much the stable isotope has been incorporated or metabolized (isotopic enrichment) was used for the final analysis, this report mainly focuses on measuring the ratios of different isotopes to accurately evaluate the technical performance and robustness of the method. This ensures that the method can reliably distinguish and measure the different isotopes. The method was validated regarding mass accuracy, linearity and working range, precision and accuracy, lower limit of quantitative differentiation (LLOQD), sample and storage stability, as well as carry-over.

### Mass accuracy

The method ought to display sufficient mass accuracy to distinguish between differently isotopically labeled metabolites ([^15^N] versus [^13^C], Suppl. Fig. 3). To determine the mass accuracy, the difference of the measured mass to the theoretical monoisotopic exact mass was calculated for three isotopologues (unlabeled, [^13^C]- and [^15^N]-labeled). The mass difference was calculated in 24 distinct sample runs for unlabeled urea, [^13^C]urea, [^15^N]urea and [^15^N_2_, ^13^C, ^18^O]urea as well as unlabeled citrulline, [^13^C]citrulline, [^15^N]citrulline and [D_5_]phenylalanine as examples for the amino acid method. Results are given as the mean difference in ppm ± rms.

The mass accuracy was determined to be at least 1.49 ± 0.06 ppm for urea and −3.71 ± 0.53 ppm for amino acid isotopologues (Suppl. Tables 3 and 4).

### Linearity and working range

As no commercially available standard for single [^15^N]-labeled urea is available, human hepatocyte cell cultures derived from induced pluripotent stem cells (iPSCs) were incubated with three different amounts (0 mM, 1 mM and 2 mM) of the ^15^NH_4_Cl tracer for 24 h and the [^15^N] isotopic ratios in the supernatant were measured^52^.

Due to the use of a cell model, the isotopic enrichment observed exceeds the maximum isotopic enrichment in human control subjects and satisfactory linearity is displayed for urea, citrulline and glutamine. No significant isotopic enrichment could be detected in glycine and glutamate as they are part of an alternative (i.e. non-urea cycle) nitrogen metabolizing route and the cells used for this experiment do not have impaired ureagenesis function. The unsatisfactory linearity of arginine originates in the low isotopic enrichment in cell experiments and the comparatively large variation in arginine measurements in general (Suppl. Fig. 4). However, as arginine enrichment only accounts for a small fraction in the calculation of the relative ureagenesis function (~0.1%) the general linearity and working range are deemed satisfactory.

### Precision and accuracy

The precision was determined as the coefficient of variation (CV) in the [^15^N] isotope ratio from four different time-points (0, 5, 8, 30 min) from a healthy subject before and after tracer ingestion, simulating four different isotope enrichment states over the expected test range, with baseline natural abundance at pre tracer ingestion and maximum enrichment after 30 min. Intra-experiment precision was calculated from six individual preparations for each time point, measured as technical triplicates on one day. Inter-experiment precision was calculated from six individual preparations for each time point, measured as technical triplicates on six different days over the course of 5 weeks.

The intra-experiment CV in plasma is <10% for all time-points and all metabolites with a maximum CV of 1.6% for urea and 6.2% for arginine (Suppl. Table 5). In DBS, all time-points for arginine as well as the baseline sample for citrulline exhibit a CV slightly above 10% (Suppl. Table 6). Inter-experiment variability in plasma was <10% for all metabolites, whereas two time-points for arginine exhibited a CV >10% in DBS (Suppl. Tables 7 and 8).

### Lower limit of quantitative differentiation (LLOQD)

The isotope ratios that are measured with this method contain an intrinsic baseline in the natural abundance of the respective stable isotope, which abolishes the need for the determination of a lower limit of detection (LLOD). The LLOQD of these highly abundant metabolites was, therefore, defined as the smallest change in isotope ratio that can reliably be distinguished from the natural abundance. The LLOQD was calculated as three times the standard deviation (i.e. critical difference) of the isotopic ratio at baseline level (before tracer intake), ascertained from the intra-experiment precision.

The LLOQD for urea is 0.0384% and 0.0594% enrichment for plasma and DBS, respectively (Suppl. Table 9). Arginine and citrulline from DBS show the highest LLOQD (0.9009% and 0.4329%), whereas the LLOQD for all other amino acids is <0.3% enrichment.

### Carry-over

Carry-over was assessed as the percentage of metabolite detected in a blank run directly after sample injections (six injections). No carry-over was detected in any blank run following sample injection with the highest calibration standard (*n* = 6).

### Sample stability

As samples were measured over the course of 5 weeks for the assessment of inter-experiment variability, it can be concluded that plasma sample stability is sufficient for at least up to 1 month, as the % CV was <10% for all metabolites. Additionally, long-term stability was determined by comparing isotopic enrichment in samples from three different healthy subjects at several time points before and after tracer ingestion, measured immediately and after 1 year storage at −80 °C (Suppl. Fig. 5 and Suppl. Table 10). Glutamate enrichment levels are decreased by 12% on average after 1 year of sample storage. All other metabolites show a difference well below 10%.

### Pre-analytical stability

Potential differences from deviations in sample processing have been investigated by comparing the [^15^N]-isotope ratio of identical samples, processed immediately (centrifugation and freezing on dry ice) and after 15 and 30 min at room temperature. Delayed sample processing of up to 30 min shows an average difference of <5% (Fig. 6 and Suppl. Table 11).

### Comparison between DBS and plasma samples

In order to compare the two matrices, blood samples from the same subject, at the same time points, were collected as described above. After collection, samples were spotted on the filter card and the remaining was centrifuged to obtain plasma. Not all patients and controls had DBS collected. We could observe that there was a correlation of 0.91 (Pearson) between the two matrices (Suppl. Fig. 7A, B).

### Isotopic enrichment and relative ureagenesis calculation (RUF)

Isotopic enrichment (*IE*) is defined as the fraction of metabolite in the sample that has become isotopically labeled and is calculated modified from Patterson and colleagues^30^, i.e.

1 $${IE}=\,100* ({F}_{t}-{F}_{0})$$

where F_t_ and F_0_ represent the isotope ratio of labeled over total (i.e. labeled plus unlabeled) signal (e.g. ^15^N/(^14^N + ^15^N)) of a given time point (F_t_) and the pre-injection base line (F_0_).

Isotopic enrichment curves from patient samples are compared to previously measured controls and interpreted graphically (Suppl. Fig. 8A) as well as arithmetically for urea, arginine, citrulline, glutamine, glycine and glutamate. Enrichment curves were normalized to account for differences in tracer concentration (i.e. deviation from 2 mg/kg of tracer given per body weight, Suppl. Fig. 8B) as well as endogenous metabolite concentration (Suppl. Fig. 8C) by normalization to mean control values of the unlabeled metabolites. Furthermore, the relative ureagenesis function can be estimated by quantitative comparison of the enrichment curves according to the following equation:

2 $${RUF}=100* \frac{{R}_{P}* {T}_{P}}{{\bar{R}}_{C}* {\bar{T}}_{C}}$$

Where *RUF* is the relative ureagenesis function, *R* is the relative amount of tracer that is metabolized through the urea cycle and T is the relative amount of absorbed and metabolized tracer over the duration of the experiment compared to the total administered amount for patients (*R*_*P*_*, T*_*P*_) and controls (*R*_*C*_*, T*_*C*_). *R* is calculated in Eq. 3, where *A* is the area under the isotopic enrichment curve for the respective metabolite over the duration of the test (120 min). *T* is calculated in Eq. 4, where *T*_*measured*_ is the amount of metabolized tracer observed as the sum of enrichment (areas under the curve) of urea, arginine, citrulline, glutamine, glutamate and glycine, and *T*_*theoretical*_ is the total amount of tracer administered.

3 $$R=\frac{({A}_{{Urea}}+{A}_{{Cit}}+{A}_{{Arg}})}{{(A}_{{Urea}}+{A}_{{Cit}}+{A}_{{Arg}}+{A}_{G\mathrm{ln}}+{A}_{{Gly}}+{A}_{{Glu}})}$$

4 $$T=100* \frac{{T}_{{measured}}}{{T}_{{theoretical}}}\,$$

Metabolites in which the total metabolite concentration shows a high variation over the duration of the test (CV > 10%) are excluded from semi-quantitative analysis and will only be interpreted graphically (Suppl. Fig. 8D).

### Statistics

Results in patient group comparisons are presented as mean ± standard deviation. For multiple group comparisons, 1-way ANOVA followed by multiple comparison testing was performed. For comparisons between 2 groups, an unpaired, 2-tailed *t*-test was used. Statistical analyses were performed using GraphPad Prism 10. Differences were considered statistically significant at *p*-values of <0.05.

## Supplementary information

Supplementary Tables

## Data Availability

Supporting data for all values underlying the data presented in the graphs are provided in the Supporting Data Values file.
