# Supplementary material for: Characterization and treatment monitoring of ureagenesis disorders using stable isotopes
Source: NPJ Metab Health Dis. Author manuscript; Available in PMC 2025 May 8. (PMC12055570; doi:10.1038/s44324-025-00051-8)
Supplement: Suppl. Mat. [file EMS205314-supplement-Suppl__Mat__.pdf]

Supplementary Tables

Supplementary Table 1: Patient data.

Patient ID	Gender	neonatal onset yes: 1; no: 0	Asympt: 0 Sympt: 1	Affected gene	Variant (s)	Age at assay (y)	NH_3 at diagnosis (µmol/L)	Treatment (only if pertinent for urea cycle function)	Additional Information
**CPS1D_1**	F	1	1	***CPS1*** (NM_001875.5)	c.2291C>T, p.(Pro764Leu); c.3935dup, p.(Met1312IlefsTer11)	0.34	289	SB 2400 mg/d; Arg 1500 mg/d; Cit 1250 mg/d	Low protein diet (1.4 g/kg/d)
**CPS1D_2**	F	0	1		c.4002G>A, p.(Glu1334=) splicing; c.4229G>A, p.(Trp1410Ter)	31.8	117	PBA 3x3 g/d or GPB 3x6.5 ml/d; Arg 12 g/d	
**CPS1D_3A**	M	1	1		c.3520C>T, p.Arg1174Ter; c.4229G>A, p.Trp1410Ter	0.95	2760	GPB 2x 0.5 ml; Cit 2x 900 mg	pre-LTX
**CPS1D_3B**						1.01			21 d post-LTx
**CPS1D_3C**						1.20		Cit 1800 ng 2x/d	71 d post- LTx
**OTCD_1**	M	0	1	***OTC*** (NM_000531.6)	c.604C>T, p.(His202Tyr)	10.7	344	PBA 3g-4g-4 g/d; Cit 3x2 g/d	
**OTCD_2**	M	0	1		c.394T>C, p.(Ser132Pro)	13.6	487	GPB 3x 2.5 ml/d; Arg 2x 1.66 g/d	
**OTCD_3**	M	0	1		c.264A>T, p.(Lys88Asn)	17.9	557	GPB 3x3 ml/d; Arg 5 g/d	
**OTCD_4**	M	0	1		c.622G>A, p.(Ala208Thr)	74.4	22	Arg 3 x1.66 g/d (88 mg/kg/d)	Low protein diet (42 g/d)
**OTCD_5^^**	M	0	1		c.622G>A, p.(Ala208Thr)	82.5	26	Arg 3x1.66 g/d (76 mg/kg/d)	Low protein diet (35 g/d)
**OTCD_6**	M	0	1		c.264A>T, p.(Lys88Asn)	3.9	42	Arg 4x800 mg/d (196 mg/kg/d)	Protein in diet: 26 g/d, 1.6 g/kg/d
**OTCD_7**	M	0	1		deep intronic c.540+265G>A	2.1	52.2	Arg 4x250 mg/d (65 mg/kg/d)	positive NBS for low Cit 3.2 cut off 7.1
**OTCD_8***	M	0	1		c.264A>T, p.(Lys88Asn)	11.3	133	Arg 3x1.6 g/d (112 mg/kg/d); SB 3x1.5 g/d (100 mg/kg/d)	Protein in diet: 38 g/d, 0.87 g/kg/d
**OTCD_9***	M	0	1		c.264A>T, p.(Lys88Asn)	14.0	102	Arg 3x1.6 g/d (82 mg/kg/d)	Protein in diet: 43 g/d, 0.6 g/kg/d
**OTCD_10**	M	0	1		c.365A>G, p.(Glu122Gly)	2.3		SB 3x 1 g/d; Arg 3x1 g/d	
**OTCD_11**	M	1	1		c.594C>A, p.(Asn198Lys)	0.77		GPB 5x0.4 ml/d; Arg 5x332 mg/d	
**OTCD_12**	M	0	1		c.394T>C, p.(Ser132Pro)	34.7	453	GPB 3x5 ml/d; Arg 3x1.66 g/d; Cit 3x1 g/d	
**OTCD_13**	M	0	1		c.216+1G>A, splicing	2.8	449	GPB 3x1.5 ml/d; Arg 2x500 mg/d; Cit 2x500 mg/d	
**OTCD_14**	M	1	1		c.386G>A, p.(Arg129His)	16.6	688	L-Arg 21% 4x15mL, NaBz 4x30mL, UCD 3 Second (formula) 9-8-8-9 g	40% of liver OTC activity; neonatal onset on day 6

**OTCD_15**	F	0	1		c.958C>T, p.(Arg320Ter)	6.9	417	GPB 3x1.5 ml/d; Arg 3x12 ml/d; Cit 3x1 g/d	
**OTCD_16**[-]	F	0	1		c.433C>T, p.(Gln145Ter)	35.9	64	-	
**OTCD_17**	F	0	1		c.2T>C; p.?	4.0	614	GPB 4x2.3 ml/d; SB 3x1.5 g/d; Cit 3x1.3 g/d	
**OTCD_18**[-]	F	0	1		c.718-1G>A, splicing	5.3	340	-	
**OTCD_19_A**						8.5		GPB 3x3.5 ml/d; SB 3x2.5 g/d; Arg 3x3 g/d; Cit 3x2 g/d	
**OTCD_19_B**	F	0	1		c.274C>T, p.(Arg92Ter)	8.6	208	GPB 3x3.5 ml/d	
**OTCD_19_C**						8.6		GPB 3x3.5 ml/d; SB 3x2.5 g/d; Arg 3x3 g/d; Cit 3x2 g/d	
**OTCD_20**	F	0	1		not identified	17.7	214	GPB 3x4.5 ml/d; SB 20% 3x12 ml/d (150 mg/kg/d); Cit 3x4.3 g/d	
**OTCD_21**[^^]	F	0	1		c.622G>A, p.(Ala208Thr)	53.0	26	Arg 3x1.6 g/d (66 mg/kg/d)	Low protein diet (45 g/d)
**OTCD_22**[#]	F	0	0		c.698C>T, p.(Ala233Val)	1.3	never HA	Arg 4x0.2 g/d	Plasma OTC activity (68% of the median of female controls)
**OTCD_23_A**[#]	F	0	0		c.698C>T, p.(Ala233Val)	0.08	never HA	-	Plasma OTC activity and 37% of the median of female controls)
**OTCD_23_B**						1.4		-	
**OTCD_24**[-]	F		0		c.433C>T, p.(Gln145Ter)	2.3		-	
**OTCD_25**[-]	F	0	0		c.718-1G>A, splicing	33.8	never HA		
**OTCD_26_A**	F	0	0		c.674C>T, p.(Pro225Leu)	34.2	51	-	Mother of OTC male - fatal neonatal onset
**OTCD_26_B**						35.1		-	
**OTCD_27**	F	0	0		c.386G>A, p.(Arg129His)	35.1	51	-	
**OTCD_28**[*]	F	0	0		c.674C>T, p.(Pro225Leu)	11.1	73	-	Mother of OTC male - fatal neonatal onset
**OTCD_29**[*]	F	0	1		c.674C>T, p.(Pro225Leu)	34.3	63	-	Mother of OTC male - fatal neonatal onset (day 5)
**ASSD_1A**	M	1	1	***ASS1*** (NM_054012.4)	c.1168G>A, p.(Gly390Arg) homozygous	0.21	2262	GPB 4x0.1 ml/d; SB 4x250 mg/d; Arg 3x600 mg/d	pre-LTX; No ASS activity in fibroblasts
**ASSD_1B**						0.38		Arg-HCl 2x800 mg/d	42 d post LTx
**ASSD_2**	M	0	0		c.535T>C, p.(Trp179Arg); c.917T>G, p.(Val306Gly)	8.6	8.8	-	
**ASSD_3**	M	0	0		c.535T>C, p.(Trp179Arg) homozygous	4.4	8.4	Arg 3x600 mg/d (98 mg/kg/d)	Protein in diet: 25 g/d, 1.3 g/kg/d
**ASSD_4**	M	0	0		c.535T>C, p.(Trp179Arg); c.827T>A, p.(Met276Lys)	4.8	never HA	Arg 3x1.66 g/d	
**ASLD_1**	F	1	1	***ASL*** (NM_000048.4)	c.470G>T, p.(Gly157Val); r.568_602del, p.(Val190TrpfsTer33)	0.16	281	GPB 4x 0.5 ml/d; Arg 118 mg/kg/d	

**ASLD_2**	F	1	1		c.707G>A, p.(Arg236Gln) homozygous	5.9	317	GPB 3x0.5 ml/d; SB 3x1g/d (170mg/kg/d); Arg 2x1.66 g + 800 mg (234 mg/kg/d)	
**ASLD_3A**	F	1	1		c.719-1G>A; splicing homozygous	0.48	810	GPB 3x0.3 ml/d; Arg 2x300 mg/d	pre-LTX
**ASLD_3B**						0.81		Arg 3 x 200 mg/d	100 d post-LTx
**ASLD_4_A**	F	1	1		c.436C>T, p.(Arg146Trp) homozygous	7.4	1261	SB 5 g/d (200 mg/kg/d); Arg 5 g/d (200 mg/kg/d)	pre-LTX
**ASLD_4_B**						8.6			1 year post-LTx
**ASLD_5A**	M	1	1		c.479A>C, p.(His160Pro) homozygous	0.87	1052	GPB 1 ml/d; Arg 3x280 mg/d	pre-LTX
**ASLD_5B**						1.1		Arg 2x300 mg/d	30 d post-LTx
**ASLD_6_A**	M	1	1		c.1128C>A, p.(Tyr376Ter) homozygous	0.25	204	SB 3x150 mg/d (80 mg/kg/d); Arg 4x250 mg/d (177 mg/kg/d)	pre-LTX
**ASLD_6_B**						1.6	193	-	6 m post-LTx
**ASLD_7A**	M	1	1		c.1366C>G, p.(Arg456Gly) homozygous	17.6	na	SB 3x4 g/d (214 mg/kg/d); Arg 4x1.66 g/d (118 mg/kg/d)	
**ASLD_7B**	M					18.1			
**ARGD_1**	M	0	1	***ARG1*** (NM_000045.4)	c.306-506A>G, r.305_306ins115, p.(Leu103LysfsTer6)	2.8	110	-	
**ARGD_2**	M	0	1		not known	7.8	not known	SB 3x500 mg/d	
**ARGD_3A**	M	1	1		c.647ins32bp, p.?; c.871C>T, p.(Arg291Ter)	13.9	>400	SB 2x1 g/d	
**ARGD_3B**						14.4		SB 2x1 g/d	Patient under ERT
**CTND_1**	F	1	1	***SLC25A13*** (NM_014251.3)	c.1628dup, p.(Ile544TyrfsTer24) homozygous	2.5	not elevated		
**CTND_2**[+]	F	0	1		c.1311+1G>A, splicing; c.1348del, p.(Glu450LysfsTer58)	11.5	not elevated	Arg 2x6 g/d; MCT 1x 15 mL/d	
**CTND_3**[+]	M	0	1		c.1311+1G>A, splicing; c.1348del, p.(Glu450LysfsTer58)	17.4	not elevated	Arg 2x6 g/d; MCT 1x 15 mL/d	
**CTND_4**	M	1	1		c.173_174del, p.(Val58GlyfsTer24); c.1813C>T, p.(Arg605Ter)	4.9	not elevated	-	
**CTND_5**	M	?	1		c.74C>A, p.(Ala25Glu); c.1078C>T, p.(Arg360Ter)	9.5	not elevated	MCT 2x5 mL/d	

**CTND_6**	M	0	1		c.1177+1G>A, p.(Ala340_Arg392del); c.1763G>A, p.(Arg588Gln)	52.8	not known	sodium pyruvate 3x2.5 g/d; L-ornithine-L-aspartate 3x6 g/d	
**HHH_1A**						0.02		PBA 250 mg/kg/d; SB 250 mg/kg/d; Arg 2 mmol/kg/d	
**HHH_1B**	M	1	1	***SLC25A15*** (NM_014252.4)	c.535C>T, p.(Arg179Ter) homozygous	0.02	910	PBA 250 mg/kg/d; SB 250 mg/kg/d; Arg 2 mmol/kg/d	
**HHH_1C**						1.8		SB 2x 350 mg/d (approx. 55 mg/kg/d); Arg 2x 500 mg/d (approx. 80 mg/kg/d)	
**HHH_2**	M	0	0		c.380C>T, p.(Thr127 Met) homozygous	9.0	not elevated		
**PA**	M	1	1	*PCCA/B*		22.3	slightly elevated	-	
**HE**	M	0	1	-	-	68.2	186	-	chronic liver disease, adult, secondary HA, single event, hepatic encephalopathy
**LPI**	M	0	1	***SLC7A7*** (NM_003982.4)	c.726G>A, (p.Trp242Ter) homozygous	9.6	57	GPB 3 x 1 ml/d; Cit 2 x 500 mg/d (50 mg/kg/d)	
**TMEM70D**	M	0	1	***TMEM70*** (NM_017866.6)	c.317-2A>G, splicing homozygous	8.3	120	-	
**DLDD**	M	0	1	***DLD*** (NM_000108.5)	c.685G >T, p.(Gly229Cys) homozygous	18.5	211	-	

Arg: L-arginine; ARGD: arginase 1 deficiency; ASLD: argininosuccinate lyase deficiency; ASSD: argininosuccinate synthetase deficiency; CD: citrin deficiency; Cit: L-citrulline; CPS1D: carbamoylphosphate synthetase 1 deficiency; DLDD: dihydrolipoamide dehydrogenase deficiency; GPB: glycerol phenylbutyrate (1.1g/ml); ERT: enzyme replacement therapy; HA: hyperammonemia; HE: hepatic encephalopathy and chronic liver disease; HHH: hyperornithinemia, hyperammonemia and homocitrullinuria syndrome; LPI: lysinuric protein intolerance; LTx: liver transplantation; MCT: medium-chain triglycerides (oil); OTC: ornithine transcarbamylase deficiency; PA: propionic acidemia; PBA: sodium phenylbutyrate or sodium phenylacetate; SB: sodium benzoate; TMEM70D: transmembrane protein 70 deficiency; na: not available; ^^: father/daughter; +: brother/sister; * mother/ daughter; ** brothers; - mother/ daughter; # aunt/niece

Supplementary Table 2: Values for the BMI, genotype severity, % of tracer recovery, % of the tracer in the UC and in alternative pathways, and plasma RUF values for all patients.

ID	Age at assay (y)	BMI (kg/m²)	Genotype Severity mild (1); intermediate (2); severe (3)	Total tracer recovered (%)	Tracer in UC (%)	Alternative Pathway (%)	RUF (%)
**CTRL (n=22)**	36.1 ±13.8	23.2 ±2.4	-	51.00 ± 10.74	99.36 ± 0.27	0.64 ± 0.27	98.95 ± 20.51
**CPS1D_1**	**0.340**	16.72	2	15.41	91.20	8.8	27.51
**CPS1D_2**	31.795	26.993	2	27.57	85.5	14.5	46.18
**CPS1D_3A**	**0.945**	14.71	3	2.34	16.3	83.8	0.75
**CPS1D_3B**	1.005	No data	3	13.30	98.2	1.8	25.58
**CPS1D_3C**	1.200	14.64	3	39.81	98.6	1.4	76.88
**OTCD_1**	10.7	21.72	2	3.2	58.1	41.9	3.7
**OTCD_2**	13.6	13.92	1	32.0	98.0	2.0	61.5
**OTCD_3**	17.9	24.91	1	50.2	99.4	0.6	97.8
**OTCD_4**	74.4	31.89	1	34.7	98.3	1.7	66.8
**OTCD_5**	82.5	21.97	1	31.3	96.1	3.9	58.9
**OTCD_6**	3.9	16.46	1	66.5	99.1	0.9	129.0
**OTCD_7**	2.07	18.36	-	39.6	98.8	1.2	76.7
**OTCD_8**	11.30	19.73	1	25.7	97.7	2.3	49.2
**OTCD_9**	14.04	19.72	1	31.7	98.2	1.8	61.0
**OTCD_10**	2.31	16.98	2	8.7	95.5	4.5	16.3
**OTCD_11**	0.77	20.98	2	28.4	98.3	1.7	54.7
**OTCD_12**	34.69	24.26	1	35.5	98.6	1.4	68.6
**OTCD_13**	2.84	16.62	2	Samples not analyzed, pre-analytical issues			
**OTCD_14**	16.64	18.63	2	21.6	97.9	2.1	41.4
**OTCD_15**	6.90	14.83	Not applicable	11.0	92.1	7.9	19.9
**OTCD_16**	35.9	23.31	Not applicable	20.2	92.8	7.2	36.8
**OTCD_17**	3.984	No data	Not applicable	14.2	89.4	10.6	24.9
**OTCD_18**	5.255	No data	Not applicable	16.2	93.3	6.7	29.6
**OTCD_19_A**	8.507	20.33	Not applicable	6.0	73.7	26.3	8.7
**OTCD_19_B**	8.562	20.84	Not applicable	8.5	83.5	16.5	13.9
**OTCD_19_C**	8.6	20.56	Not applicable	10.8	86.3	13.7	18.3
**OTCD_20**	17.7	20.13	Not applicable	10.9	89.2	10.8	19.1
**OTCD_21**	53.0	25.21	Not applicable	41.8	97.9	2.2	80.0
**OTCD_22**	1.342	15.41	Not applicable	27.4	96.4	3.6	51.7
**OTCD_23_A**	**0.077**	11.57	Not applicable	1.6	48.0	52.0	1.5
**OTCD_23_B**	1.441	13.40	Not applicable	30.4	96.8	3.2	57.5
**OTCD_24**	2.277	17.28	Not applicable	46.3	98.0	2.0	88.8
**OTCD_25**	33.8	No data	Not applicable	37.0	98.2	1.8	71.2
**OTCD_26_A**	34.2	No data	Not applicable	16.0	92.4	7.7	29.0
**OTCD_26_B**	35.1	No data	Not applicable	20.2	96.6	3.4	38.1
**OTCD_27**	35.1	No data	Not applicable	29.6	97.3	2.7	56.3
**OTCD_28**	11.1	16.30	Not applicable	17.0	94.2	5.8	31.9
**OTCD_29**	34.3	22.76	Not applicable	42.7	98.3	1.8	83.0
**ASSD_1A**	**0.208**	18.50	3	2.380	56.300	43.700	2.62
**ASSD_1B**	**0.384**	20.19	3	33.380	97.480	2.520	63.70
**ASSD_2**	8.638	20.81	1	30.330	99.800	0.200	59.26

**ASSD_3**	4.438	16.29	1	34.500	99.070	0.930	66.92
**ASSD_4**	4.800	12.93	1	23.380	99.050	0.950	45.35
**ASLD_1**	0.156	14.65	2	1.330	53.880	46.120	1.40
**ASLD_2**	5.858	17.96	2	1.270	57.030	42.970	1.42
**ASLD_3A**	0.479	15.53	3	0.950	33.120	66.880	0.62
**ASLD_3B**	0.808	11.30	3	Samples not analyzed, pre-analytical issues			
**ASLD_4_A**	7.438	16.53	3	2.430	40.940	59.060	1.94
**ASLD_4_B**	8.570	14.68	3	55.890	99.250	0.750	108.60
**ASLD_5A**	0.868	18.83	2	1.190	8.750	91.250	0.20
**ASLD_5B**	1.129	17.99	2	8.260	95.020	4.980	15.37
**ASLD_6_A**	0.252	16.65	3	0.960	32.590	67.410	0.62
**ASLD_6_B**	1.559	16.89	3	35.850	99.060	0.940	69.53
**ASLD_7A**	17.589	20.04	3	0.190	24.510	75.490	0.09
**ASLD_7B**	18.112	20.06	3	1.940	52.030	47.970	1.98
**ARGD_1**	2.803	No data	3	Only DBS data			
**ARGD_2**	7.844	15.97	3	7.490	93.190	6.810	13.67
**ARGD_3A**	13.896	No data	3	2.660	53.340	46.660	2.78
**ARGD_3B**	14.375	16.32	3	14.840	93.610	6.390	27.20
**CTND_1**	2.452	17.23	3	37.79	99.40	0.60	73.55
**CTND_2**	11.51	No data	3	63.77	99.71	0.29	124.50
**CTND_3**	17.44	No data	3	51.52	99.68	0.32	100.55
**CTND_4**	4.934	15.35	3	54.69	99.87	0.13	106.94
**CTND_5**	9.490	16.24	2	56.53	99.00	1.00	109.57
**CTND_6**	52.756	18.51	3	33.67	98.61	1.39	65.00
**HHH_1A**	**0.019**	No data	3	1.45	90.06	9.94	2.55
**HHH_1B**	**0.022**	No data	3	10.58	96.85	3.15	20.07
**HHH_1C**	1.7671	16.555	3	35.78	97.97	2.03	68.64
**HHH_2**	9.0356	No data	1	27.91	98.82	1.18	54
**PA**	22.304	15.78	-	58.570	99.330	0.670	113.910
**HE**	68.178	24.10	Not applicable	58.420	99.100	0.900	113.000
**LPI**	9.62	14.62	3	2.90	73.12	26.88	4.15
**TMEM70**	8.32	13.35	2	30.23	98.21	1.79	60.04
**DLDD**	18.45	20.09	2	41.33	99.52	0.48	80.79

ARGD: arginase 1 deficiency; ASLD: argininosuccinate lyase deficiency; ASSD: argininosuccinate synthetase deficiency; CD: citrin deficiency; Cit: L-citrulline; CPS1D: carbamoylphosphate synthetase 1 deficiency; DLDD: dihydrolipoamide dehydrogenase deficiency; HE: hepatic encephalopathy and chronic liver disease; HHH: hyperornithinemia, hyperammonemia and homocitrullinuria syndrome; LPI: lysinuric protein intolerance; OTC: ornithine transcarbamylase deficiency; PA: propionic acidemia; TMEM70D: transmembrane protein 70 deficiency; genotype severity was classified into the 3 categories “mild”, “intermediate” and “severe” (“-“ denotes lack of information on underlying genotype).

Supplementary Table 3: Selectivity for isotopically labeled urea as measured in *m/z* for the respective protonated species.

Injection	$[Urea+H]^+$ (*m/z*)	$[[^{15}N]Urea+H]^+$ (*m/z*)	$[[^{13}C]Urea+H]^+$ (*m/z*)	$[[^{15}N_2,^{13}C,^{18}O]Urea+H]^+$ (*m/z*)
1	61.04032	62.03735	62.04365	66.04195
2	61.04030	62.03734	62.04362	66.04193
3	61.04034	62.03736	62.04367	66.04197
4	61.04030	62.03727	62.04359	66.04191
5	61.04032	62.03729	62.04362	66.04194
6	61.04031	62.03729	62.0436	66.04193
7	61.04032	62.03736	62.04364	66.04195
8	61.04030	62.03735	62.04363	66.04194
9	61.04033	62.03736	62.04364	66.04196
10	61.04017	62.03717	62.04348	66.04179
11	61.04017	62.03717	62.04347	66.04178
12	61.04014	62.03715	62.04345	66.04176
13	61.04033	62.03729	62.04361	66.04194
14	61.04032	62.03729	62.04361	66.04194
15	61.04032	62.03729	62.04361	66.04194
16	61.04040	62.03738	62.0437	66.04202
17	61.04039	62.03737	62.0437	66.04202
18	61.04040	62.03738	62.04371	66.04203
19	61.04022	62.03716	62.04350	66.04182
20	61.04022	62.03717	62.04350	66.04182
21	61.04021	62.03716	62.0435	66.0418
22	61.04020	62.03718	62.0435	66.04181
23	61.04021	62.03719	62.04352	66.04181
24	61.04021	62.03719	62.04351	66.04181
Mean	61.04028	62.03727	62.04358	66.04189
Stdev	7.56E-05	8.53E-05	8.00E-05	8.39E-05
Stdev (ppm)	1.23951	1.37626	1.29027	1.27182
Theoretical Mass	61.04019	62.03722	62.043543	66.041857
**Accuracy (ppm)**	**1.49**	**0.83**	**0.67**	**0.63**
**RMS (ppm)**	**0.06**	**0.08**	**0.07**	**0.07**

Supplementary Table 4: Selectivity for isotopically labeled amino acids as measured in *m/z* for the respective protonated species.

**Injection**	**[[D$_5$]Phenylalanine+H]$^+$ (*m/z*)**	**[Citrulline+H]$^+$ (*m/z*)**	**[[^{15}N]Citrulline+H]$^+$ (*m/z*)**	**[[^{13}C]Citrulline+H]$^+$ (*m/z*)**
1	341.16537	346.15066	347.14794	347.1539
2	341.1654	346.15072	347.14778	347.15395
3	341.1654	346.15076	347.14784	347.15396
4	341.16549	346.15068	347.14804	347.15392
5	341.1654	346.15069	347.14785	347.15397
6	341.16559	346.1508	347.14794	347.15412
7	341.16536	346.15066	347.14789	347.15391
8	341.16536	346.1507	347.14775	347.15397
9	341.16539	346.15077	347.14786	347.15405
10	341.16463	346.14986	347.14731	347.15318
11	341.16444	346.14986	347.14699	347.15312
12	341.16466	346.14994	347.14715	347.15321
13	341.16518	346.15054	347.14794	347.1537
14	341.16518	346.1506	347.15379	347.14798
15	341.16532	346.15064	347.14800	347.1538
16	341.16564	346.15091	347.14812	347.15416
17	341.16559	346.1509	347.14803	347.1542
18	341.1655	346.15092	347.14801	347.15426
19	341.16532	346.15065	347.14795	347.15387
20	341.16521	346.15053	347.14777	347.15382
21	341.16522	346.1506	347.14777	347.15386
22	341.16458	346.14995	347.1473	347.15314
23	341.16459	346.1499	347.14719	347.15305
24	341.16472	346.15001	347.14722	347.15321
Mean	341.16519	346.15051	347.14798	347.15351
Stdev	0.00037	0.00036	0.00128	0.00124
Stdev (ppm)	1.08	1.05	3.69	3.57
Theoretical Mass	341.16565	346.1515	347.1485	347.1548
**Accuracy (ppm)**	**-1.36**	**-2.86**	**-1.50**	**-3.71**
**RMS (ppm)**	**0.05**	**0.05**	**0.57**	**0.53**

Supplementary Table 5: Intra-experiment variability in plasma.

Metabolite	Time (min)	Average [^{15}N] isotopic ratio(%)	Standard deviation (%)	CV (%), n=18
**[^{15}N]Urea**	0	0.7826	0.0128	**1.63**
	5	0.9687	0.0159	**1.64**
	8	1.1751	0.0187	**1.59**
	30	1.3882	0.0179	**1.29**
**[^{15}N]Arginine**	0	1.7529	0.0585	**3.34**
	5	2.0665	0.1279	**6.19**
	8	2.7192	0.1137	**4.18**
	30	3.8804	0.1170	**3.02**
**[^{15}N]Citrulline**	0	1.3997	0.0836	**5.97**
	5	4.5465	0.1679	**3.69**
	8	9.8847	0.1794	**1.82**
	30	18.4575	0.3721	**2.02**
**[^{15}N]Glutamine**	0	1.1278	0.0464	**4.12**
	5	1.2421	0.0519	**4.17**
	8	1.5772	0.0522	**3.31**
	30	2.8485	0.0513	**1.80**
**[^{15}N]Glycine**	0	0.9579	0.0276	**2.88**
	5	0.9719	0.0244	**2.52**
	8	0.9931	0.0205	**2.06**
	30	1.4914	0.0267	**1.79**
**[^{15}N]Glutamate**	0	0.7997	0.0700	**8.75**
	5	1.3550	0.0550	**4.06**
	8	2.9755	0.0744	**2.50**
	30	2.9703	0.0526	**1.77**

Supplementary Table 6: Intra-experiment variation in dried blood spots (DBS).

Metabolite	Time (min)	Average [^{15}N] isotopic ratio(%)	Standard deviation (%)	CV (%), n=18
[^{15}N]Urea	0	0.7286	0.0198	**2.72**
	5	0.9007	0.0268	**2.98**
	8	1.1105	0.0187	**1.69**
	30	1.2983	0.0295	**2.27**
[^{15}N]Arginine	0	1.2687	0.3003	**23.67**
	5	1.4821	0.2202	**14.86**
	8	1.7232	0.3078	**17.86**
	30	2.2133	0.2984	**13.48**
[^{15}N]Citrulline	0	1.3155	0.1443	**10.97**
	5	3.1285	0.1425	**4.56**
	8	6.1967	0.2475	**3.99**
	30	11.0944	0.2681	**2.42**
[^{15}N]Glutamine	0	1.0974	0.0481	**4.38**
	5	1.1895	0.0557	**4.68**
	8	1.3767	0.0448	**3.25**
	30	2.1734	0.0577	**2.65**
[^{15}N]Glycine	0	0.9660	0.0358	**3.70**
	5	0.9496	0.0422	**4.44**
	8	0.9717	0.0348	**3.59**
	30	1.2081	0.0378	**3.13**
[^{15}N]Glutamate	0	0.7788	0.0362	**4.65**
	5	0.9272	0.0692	**7.46**
	8	1.1929	0.0568	**4.76**
	30	1.1728	0.0564	**4.81**

Supplementary Table 7: Inter-experiment variability in plasma.

Metabolite	Time (min)	Average [^{15}N] isotopic ratio (%)	Standard deviation (%)	CV (%), n=18
[^{15}N]Urea	0	0.7629	0.0268	**3.51**
	5	0.9530	0.0181	**1.90**
	8	1.1542	0.0351	**3.04**
	30	1.3834	0.0299	**2.16**
[^{15}N]Arginine	0	1.6713	0.1283	**7.68**
	5	2.0200	0.1700	**8.42**
	8	2.6848	0.1198	**4.46**
	30	3.8253	0.1344	**3.51**
[^{15}N]Citrulline	0	1.4619	0.1001	**6.85**
	5	4.6607	0.1651	**3.54**
	8	10.0597	0.1981	**1.97**
	30	18.5189	0.2836	**1.53**
[^{15}N]Glutamine	0	1.1156	0.0992	**8.89**
	5	1.2753	0.0612	**4.80**
	8	1.5594	0.0513	**3.29**
	30	2.8584	0.0683	**2.39**
[^{15}N]Glycine	0	0.9497	0.0312	**3.29**
	5	0.9718	0.0266	**2.73**
	8	0.9866	0.0281	**2.84**
	30	1.5053	0.0355	**2.36**
[^{15}N]Glutamate	0	0.7829	0.0594	**7.59**
	5	1.4984	0.1089	**7.27**
	8	3.1115	0.1596	**5.13**
	30	3.0793	0.1031	**3.35**

Supplementary Table 8: Inter-experiment variability in dried blood spots (DBS).

**Metabolite**	**Time (min)**	**Average [^{15}N] isotopic ratio(%)**	**Standard deviation**	**CV (%), n=18**
**[^{15}N]Urea**	0	0.7288	0.0224	**3.07***
	5	0.9066	0.0378	**4.17***
	8	1.1051	0.0273	**2.47***
	30	1.3075	0.0435	**3.33***
**[^{15}N]Arginine**	0	1.4458	0.2147	**14.85**
	5	1.6086	0.1462	**9.09**
	8	1.9136	0.1377	**7.20**
	30	2.4129	0.2645	**10.96**
**[^{15}N]Citrulline**	0	1.3589	0.1207	**8.88**
	5	3.2278	0.1351	**4.19**
	8	6.3587	0.1989	**3.13**
	30	11.2301	0.2864	**2.55**
**[^{15}N]Glutamine**	0	1.1089	0.0364	**3.28**
	5	1.2043	0.0415	**3.45**
	8	1.4028	0.0453	**3.23**
	30	2.1991	0.0816	**3.71**
**[^{15}N]Glycine**	0	0.9469	0.0317	**3.34**
	5	0.9650	0.0248	**2.57**
	8	0.9823	0.0282	**2.87**
	30	1.1929	0.0277	**2.33**
**[^{15}N]Glutamate**	0	0.7531	0.0446	**5.92**
	5	0.9357	0.0868	**9.28**
	8	1.2012	0.0650	**5.41**
	30	1.2054	0.0389	**3.23**

*Urea measurements for DBS only contain data from four different replicates (n=12).

Supplementary Table 9: LLOQD for [^{15}N] isotopic enrichment in urea cycle metabolites.

Matrix	Metabolite	[^{15}N] Isotopic ratio (%)	Standard deviation (%)	LLOQD (%)
Plasma	**[^{15}N]Urea**	0.7826	0.0128	**0.0384**
	**[^{15}N]Arginine**	1.7529	0.0585	**0.1755**
	**[^{15}N]Citrulline**	1.3997	0.0836	**0.2508**
	**[^{15}N]Glutamine**	1.1278	0.0464	**0.1392**
	**[^{15}N]Glycine**	0.9579	0.0276	**0.0828**
	**[^{15}N]Glutamate**	0.7997	0.0700	**0.2100**
DBS	**[^{15}N]Urea**	0.7286	0.0198	**0.0594**
	**[^{15}N]Arginine**	1.2687	0.3003	**0.9009**
	**[^{15}N]Citrulline**	1.3155	0.1443	**0.4329**
	**[^{15}N]Glutamine**	1.0974	0.0481	**0.1443**
	**[^{15}N]Glycine**	0.9660	0.0358	**0.1074**
	**[15N]Glutamate**	0.7788	0.0362	**0.1086**

Supplementary Table 10: Average relative difference in [^{15}N] isotope ratio, calculated as the average difference from all time-points for each metabolite per test subject as well as cumulative over all subjects.

	**Average difference (%)**			
	**P1**	**P2**	**P3**	**Cumulative**
**Urea**	-2.83	2.19	-4.96	**-1.87**
**Citrulline**	4.42	-2.04	-2.90	**-0.17**
**Arginine**	-6.21	-0.54	-11.67	**-6.14**
**Glutamine**	0.02	1.48	-2.19	**-0.23**
**Glycine**	2.28	-0.79	-0.30	**0.40**
**Glutamate**	-12.08	-7.44	-16.65	**-12.06**

Supplementary Table 11: Average relative difference in [^{15}N] isotope ratio due to delayed sample processing.

	**Average difference (%)**	
	**15 minutes**	**30 minutes**
**[^{15}N]Urea**	0.13	1.27
**[^{15}N]Citrulline**	1.71	3.19
**[^{15}N]Arginine**	-3.86	3.13
**[^{15}N]Glutamine**	0.19	1.15
**[^{15}N]Glycine**	0.03	-0.41
**[^{15}N]Glutamate**	3.96	3.86

Supplementary Table 12: Unlabeled and isotopically labeled metabolites including their protonated mass formula and exact mass

Metabolite	Protonated Mass Formula	Protonated Exact mass (g/mol)
Urea	CH_5N_2O	61.04019
[^{13}C]urea	$^{13}CH_5N_2O$	62.04354
[^{15}N]-urea	$CH_5{}^{15}NNO$	62.03722
[$^{15}N_2$]-urea	$CH_5{}^{15}N_2O$	63.03426
IS-Urea	$^{13}CH_5{}^{15}N_2{}^{18}O$	66.04186
**Derivatized amino acids**		
Glutamine	$C_{15}H_{17}N_4O_4$	317.1249
[^{15}N]Glutamine	$C_{15}H_{17}{}^{15}NN_3O_4$	318.1220
[^{13}C]Glutamine	$^{13}CC_{14}H_{17}N_2O_4$	318.1283
Citrulline	$C_{16}H_{20}N_5O_4$	346.1515
[^{15}N]Citrulline	$C_{16}H_{20}{}^{15}NN_4O_4$	347.1485
[^{13}C]Citrulline	$^{13}CC_{15}H_{20}N_5O_4$	347.1548
Arginine	$C_{16}H_{21}N_6O_3$	345.1675
[^{15}N]Arginine	$C_{16}H_{21}{}^{15}NN_5O_3$	346.1645
[^{13}C]Arginine	$^{13}CC_{15}H_{21}N_6O_3$	346.1708
Ornithine	$C_5H_{13}N_2O_2$	133.0977
[^{15}N]Ornithine	$C_5H_{13}{}^{15}NNO_2$	134.0947
[^{13}C]Ornithine	$^{13}CC_4H_{13}N_2O_2$	134.1011
Argininosuccinic acid	$C_{20}H_{25}N_6O_7$	461.1784
[^{15}N]Argininosuccinic acid	$C_{20}H_{25}{}^{15}NN_5O_7$	462.1755
[^{13}C]Argininosuccinic acid	$^{13}CC_{19}H_{25}N_6O_7$	462.1818
Glutamate	$C_{15}H_{16}N_3O_5$	318.1090
[^{15}N]Glutamate	$C_{15}H_{16}{}^{15}NN_2O_5$	319.1060
[^{13}C]Glutamate	$^{13}CC_{14}H_{16}N_3O_5$	319.1123
Glycine	$C_{12}H_{12}N_3O_3$	246.0878
[^{15}N]Glycine	$C_{12}H_{12}{}^{15}NN_2O_3$	247.0849
[^{13}C]Glycine	$^{13}CC_{11}H_{12}N_3O_3$	247.0912
[D_5]Phenylalanine	$C_{19}D_5H_{13}N_3O_3$	341.1662

Supplementary Figures

Supplementary Figure 1: Body mass index (BMI) and tracer recovery

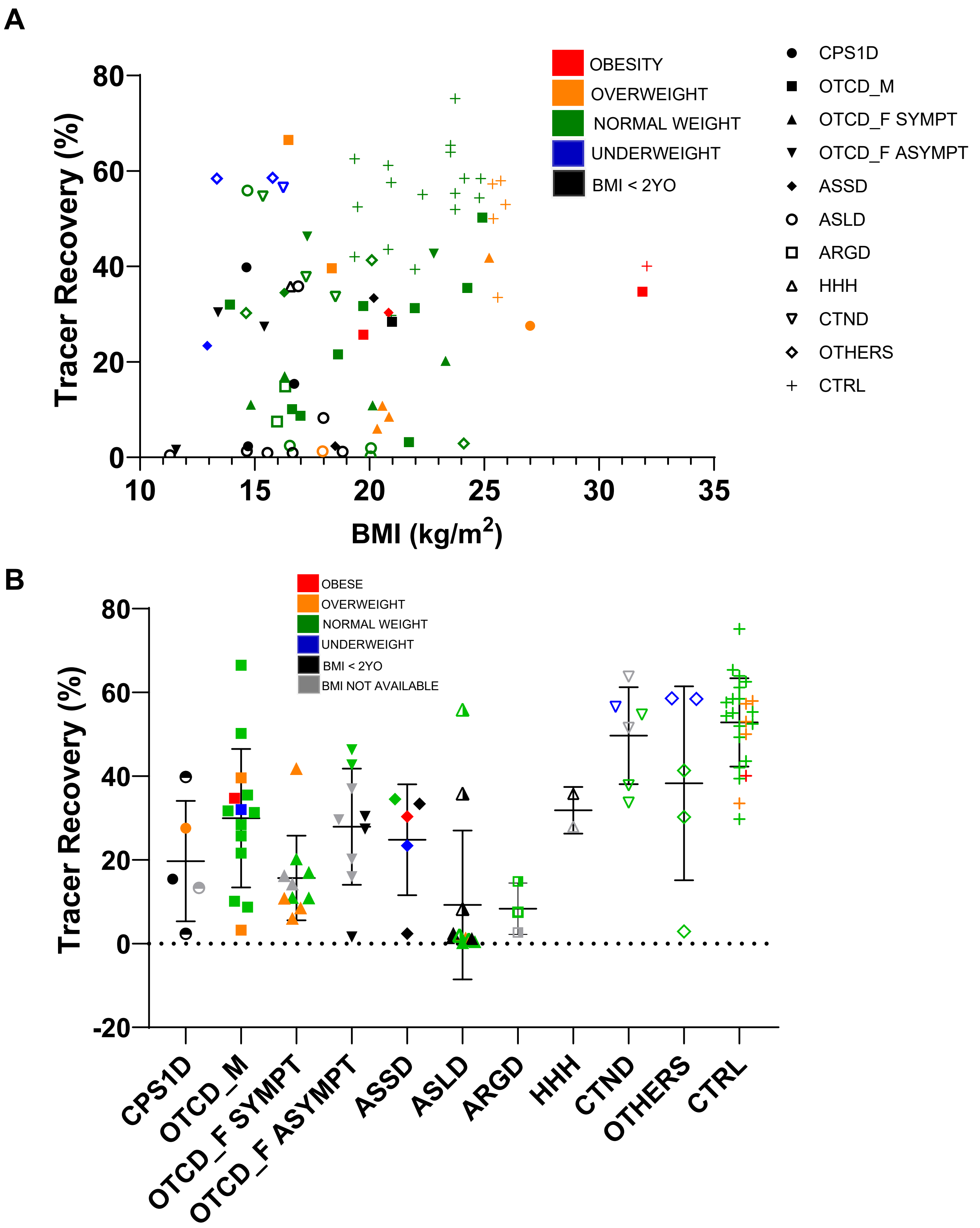

Supplementary Fig 1: **A**. Body mass index (BMI) and tracer recovery; **B**. tracer recovery per disease group. Half colored symbols represent treated patients. BMI was classified according to CDC (Center of Disease Control). Children less than 2 years-old does not have a classification for BMI.

Supplementary Figure 2: [^{15}N]glycine enrichment for citrin deficiency patients.

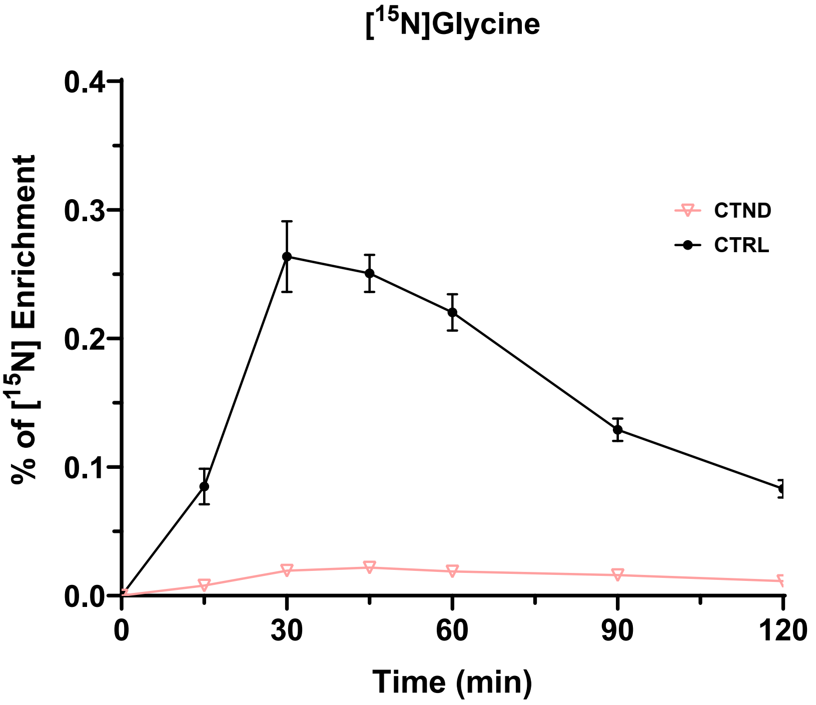

Supplementary Fig. 2: Average ± standard error of the mean of [^{15}N]glycine enrichment for citrin deficiency (CTND) patients (n=6) and controls (CTRL, in black).

Supplementary Figure 3: Separation of [^{15}N] and [^{13}C] labeled urea and citrulline

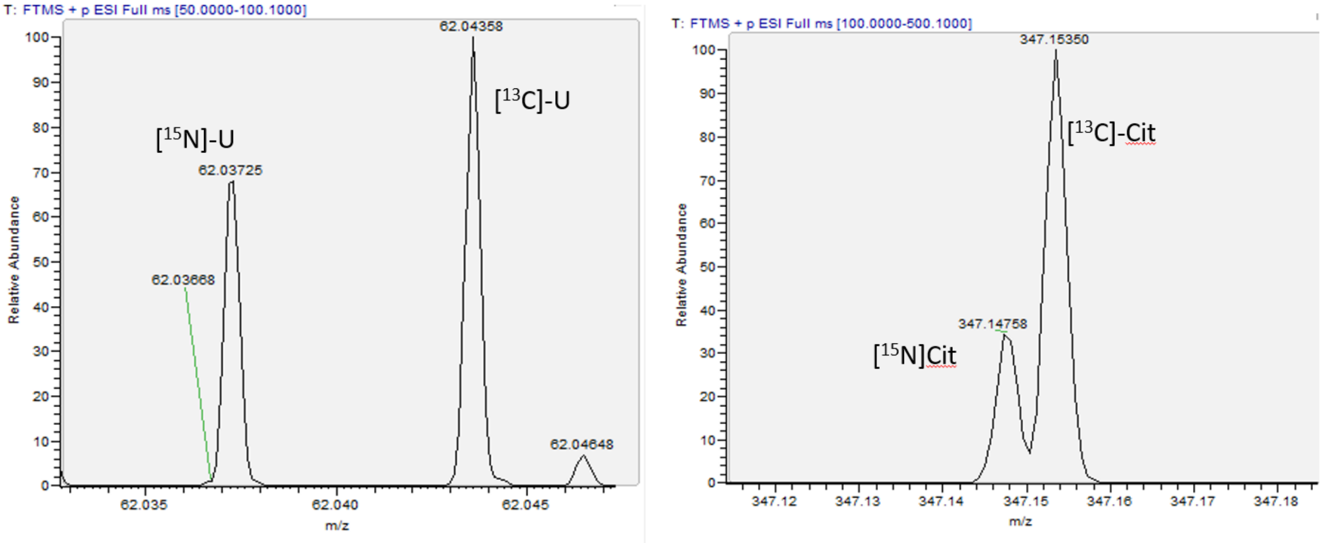

Supplementary Figure 3: Separation of [^{15}N] and [^{13}C] labeled urea and citrulline. For urea, the full scan range was set to 50.0 to 100.1 *m/z* and spectra were recorded in the profile spectrum data at a scan rate of 0.1 s/scan (maximum injection time: 0.1 s) and at a resolution of 70,000 (FWHM). For citrulline and other amino acids analysis, the full scan range was set from 100.0 to 500.1 *m/z* and spectra were recorded in profile spectrum mode at a scan rate of 0.1 s/scan (maximum injection time: 0.1 s) and at a resolution of 140,000 (FWHM).

Supplementary Figure 4: Linear isotopic enrichment

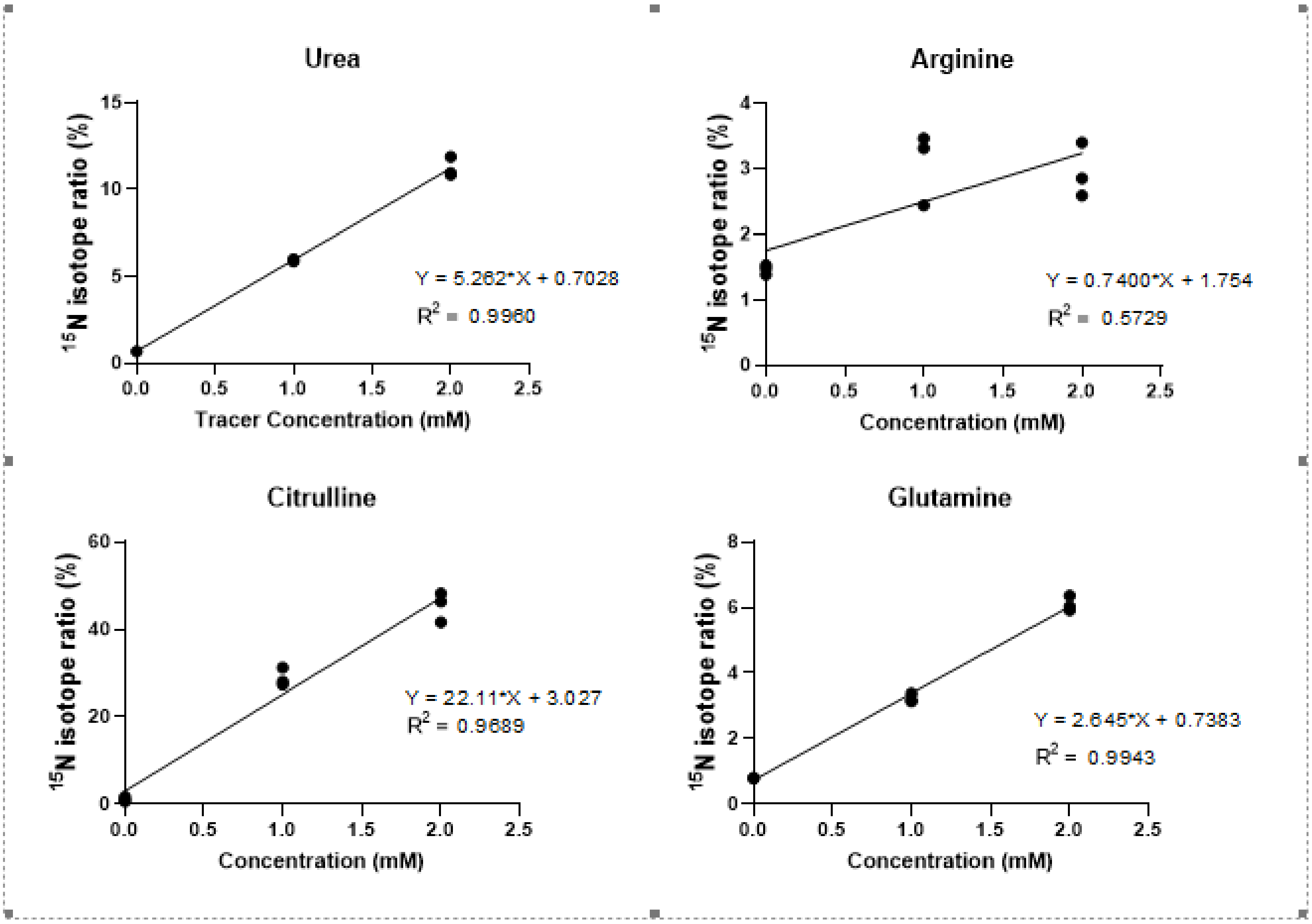

Supplementary Figure 4: Linear isotopic enrichment was obtained using human hepatocyte cell cultures derived from induced pluripotent stem cells (iPSCs) that were incubated with three different concentration (0 mM, 1 mM and 2 mM) of the $[^{15}N]H_4Cl$ tracer for 24 h in triplicates per time concentration, since no standard of $[^{15}N]$urea was commercially available.

Supplementary Figure 5: Sample stability after one year at -80°C from three different subjects

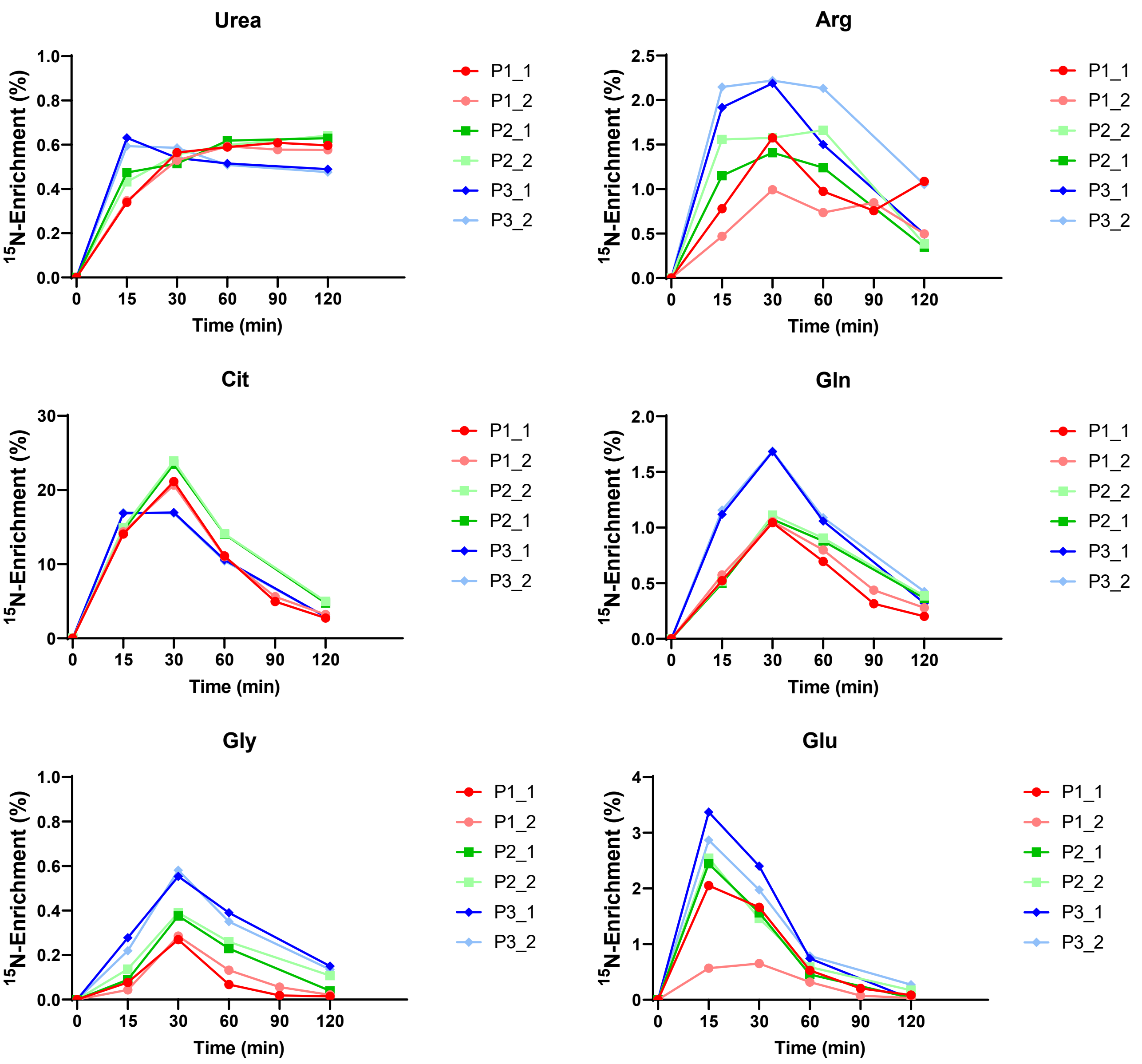

Supplementary Figure 5: Sample stability after one year at -80°C from three different subjects. Samples indexed with "_1" were immediately measured and the ones with "_2" were measured 1 year after collection.

Supplementary Figure 6: Effects of delayed sample processing on the [^{15}N] isotope ratio of ureagenesis metabolites and related amino acids

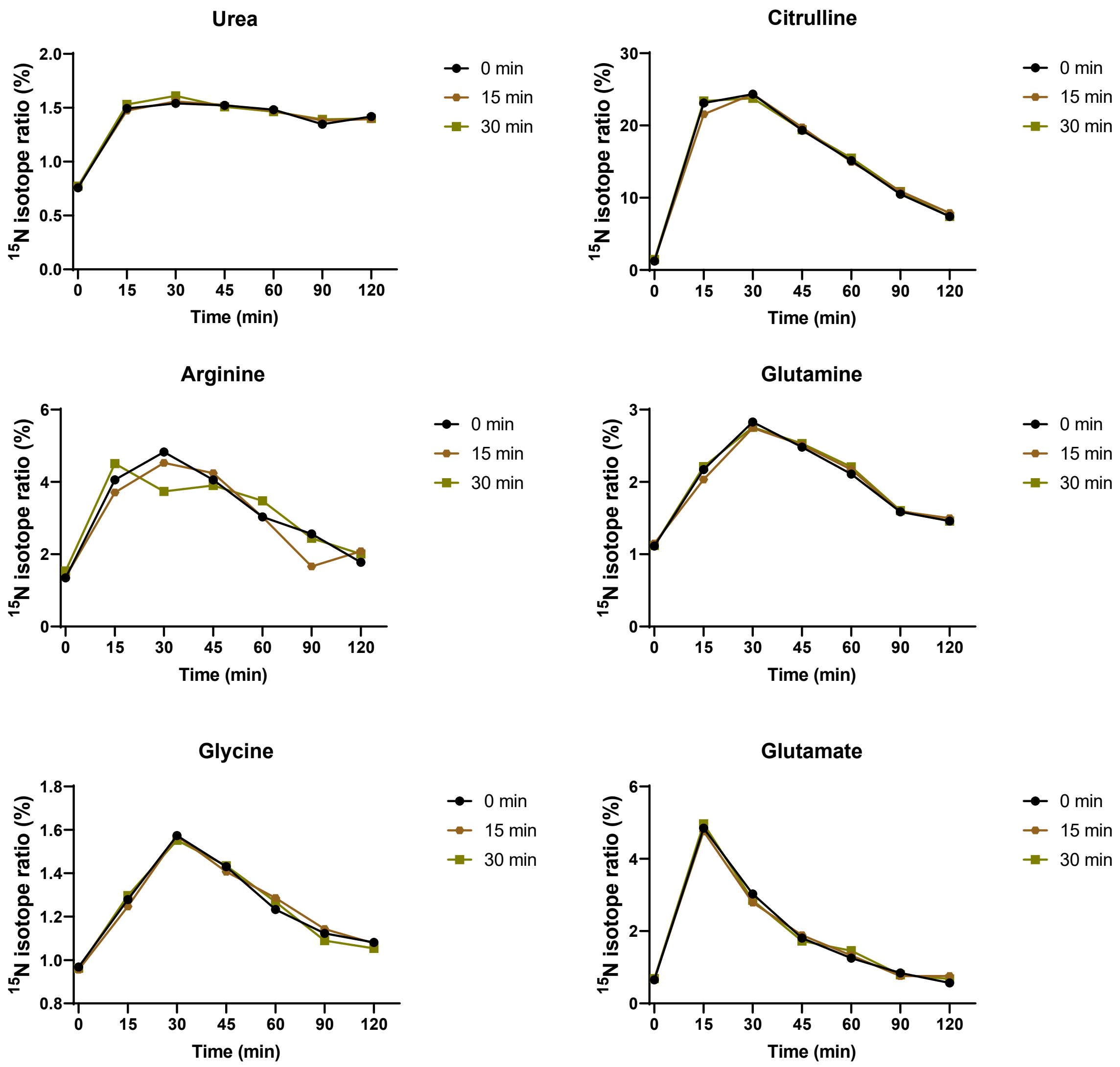

Supplementary Figure 6: Effects of delayed sample processing on the [^{15}N] isotope ratio of ureagenesis metabolites and related amino acids.

Supplementary Figure 7: Comparison of plasma and dried blood spot samples.

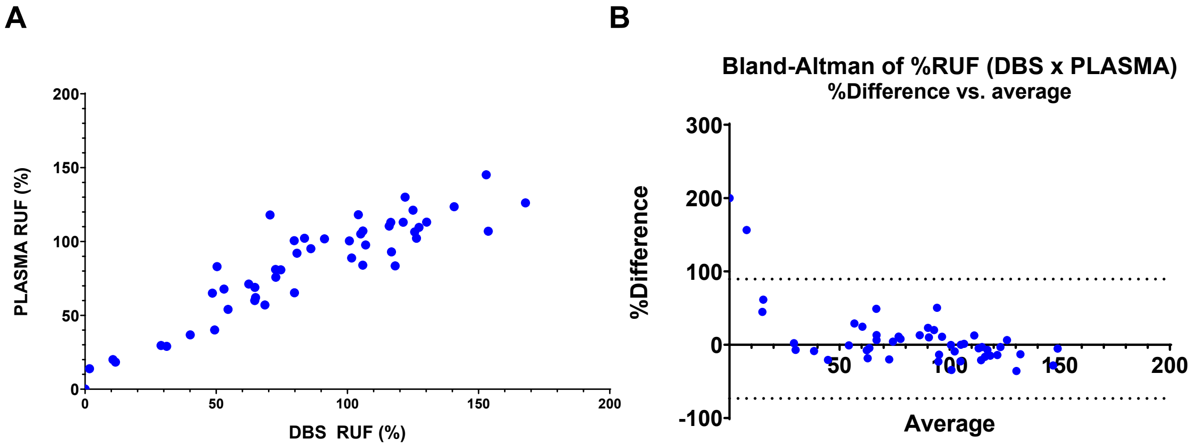

Supplementary Figure 7: **A.** Plot of samples from patients and controls that were measured in plasma and DBS (n=49). **B.** Bland-Altman plot of the %Difference vs average. The values above and below the dotted lines (95% limits of agreement) are from patients with RUF close to zero.

Supplementary Figure 8: [^{15}N] enrichment curves for urea under various conditions

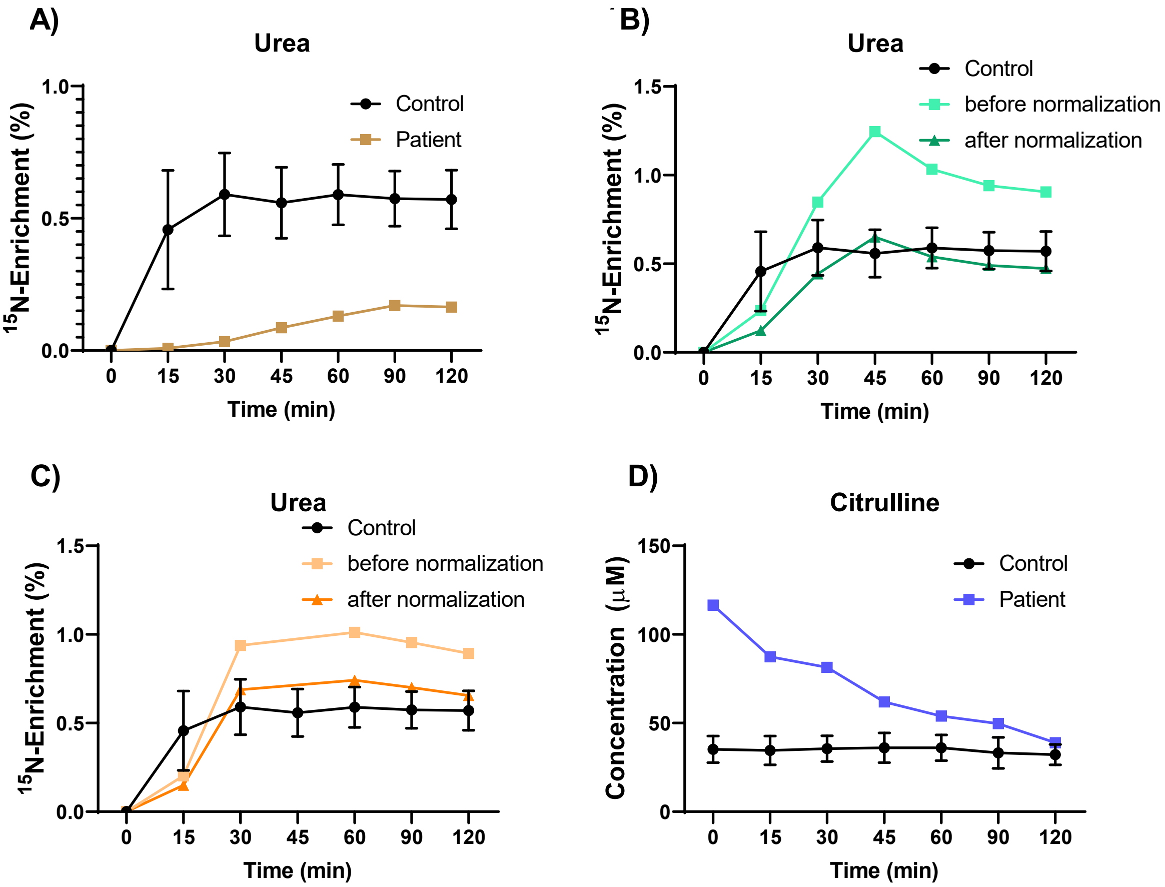

Legend to Supplementary Figure 3: Graphical analysis of [^{15}N] enrichment curves for urea for a patient with urea cycle defect (**A**). Examples of normalization to tracer amount, where the subject received the double amount of tracer (**B**), and endogenous metabolite concentration, where the subject's plasma urea concentration was 4 mM compared to 5.5 mM in controls (**C**). Example of patient (**D**) with highly variable endogenous metabolite concentration during the test, in which the results for this specific metabolite cannot be interpreted quantitatively. Controls are shown as average ± standard deviation.
