# Supplementary material for: Characterization and treatment monitoring of ureagenesis disorders using stable isotopes
Source: NPJ Metab Health Dis. 2025 May 6;3:19. doi: 10.1038/s44324-025-00051-8 (PMC12055570; doi:10.1038/s44324-025-00051-8)
Supplement: Supplementary file 1 [file 44324_2025_51_MOESM1_ESM.docx]

**Supplementary Tables**  

**Supplementary Table 1:** Patient data.

| **Patient ID** | **Gender** | **neonatal onset yes: 1; no: 0** | **Asympt: 0** **Sympt: 1** | **Affected gene** | **Variant (s)** | **Age at assay (y)** | **NH**_**3**_ **at diagnosis (µmol/L)** | **Treatment (only if pertinent for urea cycle function)** | **Additional Information** |
| --- | --- | --- | --- | --- | --- | --- | --- | --- | --- |
| **CPS1D_1** | F | 1 | 1 | ***CPS1*** (NM_001875.5) | c.2291C>T, p.(Pro764Leu); c.3935dup, p.(Met1312IlefsTer11) | 0.34 | 289 | SB 2400 mg/d; Arg 1500 mg/d; Cit 1250 mg/d | Low protein diet (1.4 g/kg/d) |
| **CPS1D_2** | F | 0 | 1 |  | c.4002G>A, p.(Glu1334=) splicing; c.4229G>A, p.(Trp1410Ter) | 31.8 | 117 | PBA 3x3 g/d or GPB 3x6.5 ml/d; Arg 12 g/d |  |
| **CPS1D_3A** | M | 1 | 1 |  | c.3520C>T, p.Arg1174Ter; c.4229G>A, p.Trp1410Ter | 0.95 | 2760 | GPB 2x 0.5 ml; Cit 2x 900 mg | pre-LTX |
| **CPS1D_3B** |  |  |  |  |  | 1.01 |  |  | 21 d post-LTx |
| **CPS1D_3C** |  |  |  |  |  | 1.20 |  | Cit 1800 ng 2x/d | 71 d post- LTx |
| **OTCD_1** | M | 0 | 1 | ***OTC*** (NM_000531.6) | c.604C>T, p.(His202Tyr) | 10.7 | 344 | PBA 3g-4g-4 g/d; Cit 3x2 g/d |  |
| **OTCD_2** | M | 0 | 1 |  | c.394T>C, p.(Ser132Pro) | 13.6 | 487 | GPB 3x 2.5 ml/d; Arg 2x 1.66 g/d |  |
| **OTCD_3** | M | 0 | 1 |  | c.264A>T, p.(Lys88Asn) | 17.9 | 557 | GPB 3x3 ml/d; Arg 5 g/d |  |
| **OTCD_4** | M | 0 | 1 |  | c.622G>A, p.(Ala208Thr) | 74.4 | 22 | Arg 3 x1.66 g/d  (88 mg/kg/d) | Low protein diet (42 g/d) |
| **OTCD_5**^**^^**^ | M | 0 | 1 |  | c.622G>A, p.(Ala208Thr) | 82.5 | 26 | Arg 3x1.66 g/d (76 mg/kg/d) | Low protein diet (35 g/d) |
| **OTCD_6** | M | 0 | 1 |  | c.264A>T, p.(Lys88Asn) | 3.9 | 42 | Arg 4x800 mg/d  (196 mg/kg/d) | Protein in diet: 26 g/d, 1.6 g/kg/d |
| **OTCD_7** | M | 0 | 1 |  | deep intronic c.540+265G>A | 2.1 | 52.2 | Arg 4x250 mg/d (65 mg/kg/d) | positive NBS for low Cit 3.2 cut off 7.1 |
| **OTCD_8*** | M | 0 | 1 |  | c.264A>T, p.(Lys88Asn) | 11.3 | 133 | Arg 3x1.6 g/d (112 mg/kg/d); SB 3x1.5 g/d (100 mg/kg/d) | Protein in diet: 38 g/d, 0.87 g/kg/d |
| **OTCD_9*** | M | 0 | 1 |  | c.264A>T, p.(Lys88Asn) | 14.0 | 102 | Arg 3x1.6 g/d (82 mg/kg/d) | Protein in diet: 43 g/d, 0.6 g/kg/d |
| **OTCD_10** | M | 0 | 1 |  | c.365A>G, p.(Glu122Gly) | 2.3 |  | SB 3x 1 g/d; Arg 3x1 g/d |  |
| **OTCD_11** | M | 1 | 1 |  | c.594C>A, p.(Asn198Lys) | 0.77 |  | GPB 5x0.4 ml/d; Arg 5x332 mg/d |  |
| **OTCD_12** | M | 0 | 1 |  | c.394T>C, p.(Ser132Pro) | 34.7 | 453 | GPB 3x5 ml/d; Arg 3x1.66 g/d; Cit 3x1 g/d |  |
| **OTCD_13** | M | 0 | 1 |  | c.216+1G>A, splicing | 2.8 | 449 | GPB 3x1.5 ml/d; Arg 2x500 mg/d; Cit 2x500 mg/d |  |
| **OTCD_14** | M | 1 | 1 |  | c.386G>A, p.(Arg129His) | 16.6 | 688 | L-Arg 21% 4x15mL, NaBz 4x30mL, UCD 3 Second (formula) 9-8-8-9 g | 40% of liver OTC activity; neonatal onset on day 6 |
| **OTCD_15** | F | 0 | 1 |  | c.958C>T, p.(Arg320Ter) | 6.9 | 417 | GPB 3x1.5 ml/d; Arg 3x12 ml/d; Cit 3x1 g/d |  |
| **OTCD_16**^**-**^ | F | 0 | 1 |  | c.433C>T, p.(Gln145Ter) | 35.9 | 64 | - |  |
| **OTCD_17** | F | 0 | 1 |  | c.2T>C; p.? | 4.0 | 614 | GPB 4x2.3 ml/d; SB 3x1.5 g/d; Cit 3x1.3 g/d |  |
| **OTCD_18**^**-**^ | F | 0 | 1 |  | c.718-1G>A, splicing | 5.3 | 340 | - |  |
| **OTCD_19_A** | F | 0 | 1 |  | c.274C>T, p.(Arg92Ter) | 8.5 | 208 | GPB 3x3.5 ml/d; SB 3x2.5 g/d; Arg 3x3 g/d; Cit 3x2 g/d |  |
| **OTCD_19_B** |  |  |  |  |  | 8.6 |  | GPB 3x3.5 ml/d |  |
| **OTCD_19_C** |  |  |  |  |  | 8.6 |  | GPB 3x3.5 ml/d; SB 3x2.5 g/d; Arg 3x3 g/d; Cit 3x2 g/d |  |
| **OTCD_20** | F | 0 | 1 |  | not identified | 17.7 | 214 | GPB 3x4.5 ml/d; SB 20% 3x12 ml/d (150 mg/kg/d); Cit 3x4.3 g/d |  |
| **OTCD_21**^**^^**^ | F | 0 | 1 |  | c.622G>A, p.(Ala208Thr) | 53.0 | 26 | Arg 3x1.6 g/d (66 mg/kg/d) | Low protein diet (45 g/d) |
| **OTCD_22**^**#**^ | F | 0 | 0 |  | c.698C>T, p.(Ala233Val) | 1.3 | never HA | Arg 4x0.2 g/d | Plasma OTC activity (68% of the median of female controls) |
| **OTCD_23_A**^**#**^ | F | 0 | 0 |  | c.698C>T, p.(Ala233Val) | 0.08 | never HA | - | Plasma OTC activity and 37% of the median of female controls) |
| **OTCD_23_B** |  |  |  |  |  | 1.4 |  | - |  |
| **OTCD_24**^**-**^ | F |  | 0 |  | c.433C>T, p.(Gln145Ter) | 2.3 |  | - |  |
| **OTCD_25**^**-**^ | F | 0 | 0 |  | c.718-1G>A, splicing | 33.8 | never HA |  |  |
| **OTCD_26_A** | F | 0 | 0 |  | c.674C>T, p.(Pro225Leu) | 34.2 | 51 | - | Mother of OTC male - fatal neonatal onset |
| **OTCD_26_B** |  |  |  |  |  | 35.1 |  | - |  |
| **OTCD_27** | F | 0 | 0 |  | c.386G>A, p.(Arg129His) | 35.1 | 51 | - |  |
| **OTCD_28**^*****^ | F | 0 | 0 |  | c.674C>T, p.(Pro225Leu) | 11.1 | 73 | - | Mother of OTC male - fatal neonatal onset |
| **OTCD_29**^*****^ | F | 0 | 1 |  | c.674C>T, p.(Pro225Leu) | 34.3 | 63 | - | Mother of OTC male - fatal neonatal onset (day 5) |
| **ASSD_1A** | M | 1 | 1 | ***ASS1*** (NM_054012.4) | c.1168G>A, p.(Gly390Arg) homozygous | 0.21 | 2262 | GPB 4x0.1 ml/d; SB 4x250 mg/d; Arg 3x600 mg/d | pre-LTX; No ASS activity in fibroblasts |
| **ASSD_1B** |  |  |  |  |  | 0.38 |  | Arg-HCl 2x800 mg/d | 42 d post LTx |
| **ASSD_2** | M | 0 | 0 |  | c.535T>C, p.(Trp179Arg); c.917T>G, p.(Val306Gly) | 8.6 | 8.8 | - |  |
| **ASSD_3** | M | 0 | 0 |  | c.535T>C, p.(Trp179Arg) homozygous | 4.4 | 8.4 | Arg 3x600 mg/d (98 mg/kg/d) | Protein in diet: 25 g/d, 1.3 g/kg/d |
| **ASSD_4** | M | 0 | 0 |  | c.535T>C, p.(Trp179Arg); c.827T>A, p.(Met276Lys) | 4.8 | never HA | Arg 3x1.66 g/d |  |
| **ASLD_1** | F | 1 | 1 | ***ASL*** (NM_000048.4) | c.470G>T, p.(Gly157Val); r.568_602del, p.(Val190TrpfsTer33) | 0.16 | 281 | GPB 4x 0.5 ml/d; Arg 118 mg/kg/d |  |
| **ASLD_2** | F | 1 | 1 |  | c.707G>A, p.(Arg236Gln) homozygous | 5.9 | 317 | GPB 3x0.5 ml/d; SB 3x1g/d (170mg/kg/d); Arg 2x1.66 g + 800 mg (234 mg/kg/d) |  |
| **ASLD_3A** | F | 1 | 1 |  | c.719-1G>A; splicing homozygous | 0.48 | 810 | GPB 3x0.3 ml/d; Arg 2x300 mg/d | pre-LTX |
| **ASLD_3B** |  |  |  |  |  | 0.81 |  | Arg 3 x 200 mg/d | 100 d post-LTx |
| **ASLD_4_A** | F | 1 | 1 |  | c.436C>T, p.(Arg146Trp)  homozygous | 7.4 | 1261 | SB 5 g/d (200 mg/kg/d); Arg 5 g/d (200 mg/kg/d) | pre-LTX |
| **ASLD_4_B** |  |  |  |  |  | 8.6 |  |  | 1 year post-LTx |
| **ASLD_5A** | M | 1 | 1 |  | c.479A>C, p.(His160Pro) homozygous | 0.87 | 1052 | GPB 1 ml/d; Arg 3x280 mg/d | pre-LTX |
| **ASLD_5B** |  |  |  |  |  | 1.1 |  | Arg 2x300 mg/d | 30 d post-LTx |
| **ASLD_6_A** | M | 1 | 1 |  | c.1128C>A, p.(Tyr376Ter) homozygous | 0.25 | 204 | SB 3x150 mg/d (80 mg/kg/d); Arg 4x250 mg/d (177 mg/kg/d) | pre-LTX |
| **ASLD_6_B** |  |  |  |  |  | 1.6 | 193 | - | 6 m post-LTx |
| **ASLD_7A** | M | 1 | 1 |  | c.1366C>G, p.(Arg456Gly) homozygous | 17.6 | na | SB 3x4 g/d (214 mg/kg/d); Arg 4x1.66 g/d (118 mg/kg/d) |  |
| **ASLD_7B** | M |  |  |  |  | 18.1 |  |  |  |
| **ARGD_1** | M | 0 | 1 | ***ARG1*** (NM_000045.4) | c.306-506A>G, r.305_306ins115, p.(Leu103LysfsTer6) | 2.8 | 110 | - |  |
| **ARGD_2** | M | 0 | 1 |  | not known | 7.8 | not known | SB 3x500 mg/d |  |
| **ARGD_3A** | M | 1 | 1 |  | c.647ins32bp, p.?; c.871C>T, p.(Arg291Ter) | 13.9 | >400 | SB 2x1 g/d |  |
| **ARGD_3B** |  |  |  |  |  | 14.4 |  | SB 2x1 g/d | Patient under ERT |
| **CTND_1** | F | 1 | 1 | ***SLC25A13*** (NM_014251.3) | c.1628dup,  p.(Ile544TyrfsTer24) homozygous | 2.5 | not elevated |  |  |
| **CTND_2**^**+**^ | F | 0 | 1 |  | c.1311+1G>A, splicing; c.1348del, p.(Glu450LysfsTer58) | 11.5 | not elevated | Arg 2x6 g/d; MCT 1x 15 mL/d |  |
| **CTND_3**^**+**^ | M | 0 | 1 |  | c.1311+1G>A, splicing; c.1348del, p.(Glu450LysfsTer58) | 17.4 | not elevated | Arg 2x6 g/d; MCT 1x 15 mL/d |  |
| **CTND_4** | M | 1 | 1 |  | c.173_174del, p.(Val58GlyfsTer24); c.1813C>T, p.(Arg605Ter) | 4.9 | not elevated | - |  |
| **CTND_5** | M | ? | 1 |  | c.74C>A, p.(Ala25Glu);  c.1078C>T, p.(Arg360Ter) | 9.5 | not elevated | MCT 2x5 mL/d |  |
| **CTND_6** | M | 0 | 1 |  | c.1177+1G>A, p.(Ala340_Arg392del);  c.1763G>A, p.(Arg588Gln) | 52.8 | not known | sodium pyruvate 3x2.5 g/d; L-ornithine-L-aspartate 3x6 g/d |  |
| **HHH_1A** | M | 1 | 1 | ***SLC25A15*** (NM_014252.4) | c.535C>T, p.(Arg179Ter) homozygous | 0.02 | 910 | PBA 250 mg/kg/d; SB 250 mg/kg/d; Arg 2 mmol/kg/d |  |
| **HHH_1B** |  |  |  |  |  | 0.02 |  | PBA 250 mg/kg/d; SB 250 mg/kg/d; Arg 2 mmol/kg/d |  |
| **HHH_1C** |  |  |  |  |  | 1.8 |  | SB 2x 350 mg/d (approx. 55 mg/kg/d); Arg 2x 500 mg/d (approx. 80 mg/kg/d) |  |
| **HHH_2** | M | 0 | 0 |  | c.380C>T, p.(Thr127 Met) homozygous | 9.0 | not elevated |  |  |
| **PA** | M | 1 | 1 | *PCCA/B* |  | 22.3 | slightly elevated | - |  |
| **HE** | M | 0 | 1 | - | - | 68.2 | 186 | - | chronic liver disease, adult, secondary HA, single event, hepatic encephalopathy |
| **LPI** | M | 0 | 1 | ***SLC7A7*** (NM_003982.4) | c.726G>A, (p.Trp242Ter) homozygous | 9.6 | 57 | GPB 3 x 1 ml/d; Cit 2 x 500 mg/d (50 mg/kg/d) |  |
| **TMEM70D** | M | 0 | 1 | ***TMEM70*** (NM_017866.6) | c.317-2A>G, splicing homozygous | 8.3 | 120 | - |  |
| **DLDD** | M | 0 | 1 | ***DLD*** (NM_000108.5) | c.685G >T, p.(Gly229Cys) homozygous | 18.5 | 211 | - |  |

Arg: L-arginine; ARGD: arginase 1 deficiency; ASLD: argininosuccinate lyase deficiency; ASSD: argininosuccinate synthetase deficiency; CD: citrin deficiency; Cit: L-citrulline; CPS1D: carbamoylphosphate synthetase 1 deficiency; DLDD: dihydrolipoamide dehydrogenase deficiency; GPB: glycerol phenylbutyrate (1.1g/ml); ERT: enzyme replacement therapy; HA: hyperammonemia; HE:  hepatic encephalopathy and chronic liver disease; HHH: hyperornithinemia, hyperammonemia and homocitrullinuria syndrome; LPI: lysinuric protein intolerance; LTx: liver transplantation; MCT: medium-chain triglycerides (oil); OTC: ornithine transcarbamylase deficiency; PA: propionic acidemia; PBA: sodium phenylbutyrate or sodium phenylacetate; SB: sodium benzoate; TMEM70D: transmembrane protein 70 deficiency; na: not available; ^^: father/daughter; +: brother/sister;  * mother/ daughter; ** brothers; - mother/ daughter; # aunt/niece

**Supplementary Table 2:** Values for the BMI, genotype severity, % of tracer recovery, % of the tracer in the UC and in alternative pathways, and plasma RUF values for all patients.

| **ID** | **Age at assay (y)** | **BMI (kg/m**^**2**^**)** | **Genotype Severity             mild (1); intermediate (2); severe (3)** | **Total tracer recovered (%)** | **Tracer in UC (%)** | **Alternative Pathway (%)** | **RUF (%)** |
| --- | --- | --- | --- | --- | --- | --- | --- |
| **CTRL (n=22)** | 36.1 ±13.8 | 23.2 ±2.4 | - | 51.00 ± 10.74 | 99.36 ± 0.27 | 0.64 ± 0.27 | 98.95 ± 20.51 |
| **CPS1D_1** | **0.340** | 16.72 | 2 | 15.41 | 91.20 | 8.8 | 27.51 |
| **CPS1D_2** | 31.795 | 26.993 | 2 | 27.57 | 85.5 | 14.5 | 46.18 |
| **CPS1D_3A** | **0.945** | 14.71 | 3 | 2.34 | 16.3 | 83.8 | 0.75 |
| **CPS1D_3B** | 1.005 | No data | 3 | 13.30 | 98.2 | 1.8 | 25.58 |
| **CPS1D_3C** | 1.200 | 14.64 | 3 | 39.81 | 98.6 | 1.4 | 76.88 |
| **OTCD_1** | 10.7 | 21.72 | 2 | 3.2 | 58.1 | 41.9 | 3.7 |
| **OTCD_2** | 13.6 | 13.92 | 1 | 32.0 | 98.0 | 2.0 | 61.5 |
| **OTCD_3** | 17.9 | 24.91 | 1 | 50.2 | 99.4 | 0.6 | 97.8 |
| **OTCD_4** | 74.4 | 31.89 | 1 | 34.7 | 98.3 | 1.7 | 66.8 |
| **OTCD_5** | 82.5 | 21.97 | 1 | 31.3 | 96.1 | 3.9 | 58.9 |
| **OTCD_6** | 3.9 | 16.46 | 1 | 66.5 | 99.1 | 0.9 | 129.0 |
| **OTCD_7** | 2.07 | 18.36 | - | 39.6 | 98.8 | 1.2 | 76.7 |
| **OTCD_8** | 11.30 | 19.73 | 1 | 25.7 | 97.7 | 2.3 | 49.2 |
| **OTCD_9** | 14.04 | 19.72 | 1 | 31.7 | 98.2 | 1.8 | 61.0 |
| **OTCD_10** | 2.31 | 16.98 | 2 | 8.7 | 95.5 | 4.5 | 16.3 |
| **OTCD_11** | 0.77 | 20.98 | 2 | 28.4 | 98.3 | 1.7 | 54.7 |
| **OTCD_12** | 34.69 | 24.26 | 1 | 35.5 | 98.6 | 1.4 | 68.6 |
| **OTCD_13** | 2.84 | 16.62 | 2 | Samples not analyzed, pre-analytical issues |  |  |  |
| **OTCD_14** | 16.64 | 18.63 | 2 | 21.6 | 97.9 | 2.1 | 41.4 |
| **OTCD_15** | 6.90 | 14.83 | Not applicable | 11.0 | 92.1 | 7.9 | 19.9 |
| **OTCD_16** | 35.9 | 23.31 | Not applicable | 20.2 | 92.8 | 7.2 | 36.8 |
| **OTCD_17** | 3.984 | No data | Not applicable | 14.2 | 89.4 | 10.6 | 24.9 |
| **OTCD_18** | 5.255 | No data | Not applicable | 16.2 | 93.3 | 6.7 | 29.6 |
| **OTCD_19_A** | 8.507 | 20.33 | Not applicable | 6.0 | 73.7 | 26.3 | 8.7 |
| **OTCD_19_B** | 8.562 | 20.84 | Not applicable | 8.5 | 83.5 | 16.5 | 13.9 |
| **OTCD_19_C** | 8.6 | 20.56 | Not applicable | 10.8 | 86.3 | 13.7 | 18.3 |
| **OTCD_20** | 17.7 | 20.13 | Not applicable | 10.9 | 89.2 | 10.8 | 19.1 |
| **OTCD_21** | 53.0 | 25.21 | Not applicable | 41.8 | 97.9 | 2.2 | 80.0 |
| **OTCD_22** | 1.342 | 15.41 | Not applicable | 27.4 | 96.4 | 3.6 | 51.7 |
| **OTCD_23_A** | **0.077** | 11.57 | Not applicable | 1.6 | 48.0 | 52.0 | 1.5 |
| **OTCD_23_B** | 1.441 | 13.40 | Not applicable | 30.4 | 96.8 | 3.2 | 57.5 |
| **OTCD_24** | 2.277 | 17.28 | Not applicable | 46.3 | 98.0 | 2.0 | 88.8 |
| **OTCD_25** | 33.8 | No data | Not applicable | 37.0 | 98.2 | 1.8 | 71.2 |
| **OTCD_26_A** | 34.2 | No data | Not applicable | 16.0 | 92.4 | 7.7 | 29.0 |
| **OTCD_26_B** | 35.1 | No data | Not applicable | 20.2 | 96.6 | 3.4 | 38.1 |
| **OTCD_27** | 35.1 | No data | Not applicable | 29.6 | 97.3 | 2.7 | 56.3 |
| **OTCD_28** | 11.1 | 16.30 | Not applicable | 17.0 | 94.2 | 5.8 | 31.9 |
| **OTCD_29** | 34.3 | 22.76 | Not applicable | 42.7 | 98.3 | 1.8 | 83.0 |
| **ASSD_1A** | **0.208** | 18.50 | 3 | 2.380 | 56.300 | 43.700 | 2.62 |
| **ASSD_1B** | **0.384** | 20.19 | 3 | 33.380 | 97.480 | 2.520 | 63.70 |
| **ASSD_2** | 8.638 | 20.81 | 1 | 30.330 | 99.800 | 0.200 | 59.26 |
| **ASSD_3** | 4.438 | 16.29 | 1 | 34.500 | 99.070 | 0.930 | 66.92 |
| **ASSD_4** | 4.800 | 12.93 | 1 | 23.380 | 99.050 | 0.950 | 45.35 |
| **ASLD_1** | 0.156 | 14.65 | 2 | 1.330 | 53.880 | 46.120 | 1.40 |
| **ASLD_2** | 5.858 | 17.96 | 2 | 1.270 | 57.030 | 42.970 | 1.42 |
| **ASLD_3A** | 0.479 | 15.53 | 3 | 0.950 | 33.120 | 66.880 | 0.62 |
| **ASLD_3B** | 0.808 | 11.30 | 3 | Samples not analyzed, pre-analytical issues |  |  |  |
| **ASLD_4_A** | 7.438 | 16.53 | 3 | 2.430 | 40.940 | 59.060 | 1.94 |
| **ASLD_4_B** | 8.570 | 14.68 | 3 | 55.890 | 99.250 | 0.750 | 108.60 |
| **ASLD_5A** | 0.868 | 18.83 | 2 | 1.190 | 8.750 | 91.250 | 0.20 |
| **ASLD_5B** | 1.129 | 17.99 | 2 | 8.260 | 95.020 | 4.980 | 15.37 |
| **ASLD_6_A** | 0.252 | 16.65 | 3 | 0.960 | 32.590 | 67.410 | 0.62 |
| **ASLD_6_B** | 1.559 | 16.89 | 3 | 35.850 | 99.060 | 0.940 | 69.53 |
| **ASLD_7A** | 17.589 | 20.04 | 3 | 0.190 | 24.510 | 75.490 | 0.09 |
| **ASLD_7B** | 18.112 | 20.06 | 3 | 1.940 | 52.030 | 47.970 | 1.98 |
| **ARGD_1** | 2.803 | No data | 3 | Only DBS data |  |  |  |
| **ARGD_2** | 7.844 | 15.97 | 3 | 7.490 | 93.190 | 6.810 | 13.67 |
| **ARGD_3A** | 13.896 | No data | 3 | 2.660 | 53.340 | 46.660 | 2.78 |
| **ARGD_3B** | 14.375 | 16.32 | 3 | 14.840 | 93.610 | 6.390 | 27.20 |
| **CTND_1** | 2.452 | 17.23 | 3 | 37.79 | 99.40 | 0.60 | 73.55 |
| **CTND_2** | 11.51 | No data | 3 | 63.77 | 99.71 | 0.29 | 124.50 |
| **CTND_3** | 17.44 | No data | 3 | 51.52 | 99.68 | 0.32 | 100.55 |
| **CTND_4** | 4.934 | 15.35 | 3 | 54.69 | 99.87 | 0.13 | 106.94 |
| **CTND_5** | 9.490 | 16.24 | 2 | 56.53 | 99.00 | 1.00 | 109.57 |
| **CTND_6** | 52.756 | 18.51 | 3 | 33.67 | 98.61 | 1.39 | 65.00 |
| **HHH_1A** | **0.019** | No data | 3 | 1.45 | 90.06 | 9.94 | 2.55 |
| **HHH_1B** | **0.022** | No data | 3 | 10.58 | 96.85 | 3.15 | 20.07 |
| **HHH_1C** | 1.7671 | 16.555 | 3 | 35.78 | 97.97 | 2.03 | 68.64 |
| **HHH_2** | 9.0356 | No data | 1 | 27.91 | 98.82 | 1.18 | 54 |
| **PA** | 22.304 | 15.78 | - | 58.570 | 99.330 | 0.670 | 113.910 |
| **HE** | 68.178 | 24.10 | Not applicable | 58.420 | 99.100 | 0.900 | 113.000 |
| **LPI** | 9.62 | 14.62 | 3 | 2.90 | 73.12 | 26.88 | 4.15 |
| **TMEM70** | 8.32 | 13.35 | 2 | 30.23 | 98.21 | 1.79 | 60.04 |
| **DLDD** | 18.45 | 20.09 | 2 | 41.33 | 99.52 | 0.48 | 80.79 |

ARGD: arginase 1 deficiency; ASLD: argininosuccinate lyase deficiency; ASSD: argininosuccinate synthetase deficiency; CD: citrin deficiency; Cit: L-citrulline; CPS1D: carbamoylphosphate synthetase 1 deficiency; DLDD: dihydrolipoamide dehydrogenase deficiency; HE: hepatic encephalopathy and chronic liver disease; HHH: hyperornithinemia, hyperammonemia and homocitrullinuria syndrome; LPI: lysinuric protein intolerance; OTC: ornithine transcarbamylase deficiency; PA: propionic acidemia; TMEM70D: transmembrane protein 70 deficiency; genotype severity was classified into the 3 categories “mild”, “intermediate” and “severe” (“-“ denotes lack of information on underlying genotype). 

**Supplementary Table 3:** Selectivity for isotopically labeled urea as measured in m/z for the respective protonated species.

| **Injection** | **[Urea+H]**^**+**^ **(*****m/z*****)** | **[[**^**15**^**N]Urea+H]**^**+**^  **(*****m/z*****)** | **[[**^**13**^**C]Urea+H]**^**+**^   **(*****m/z*****)** | **[[**^**15**^**N**_**2**_**,**^**13**^**C,**^**18**^**O]Urea+H]**^**+**^   **(*****m/z*****)** |
| --- | --- | --- | --- | --- |
| 1 | 61.04032 | 62.03735 | 62.04365 | 66.04195 |
| 2 | 61.04030 | 62.03734 | 62.04362 | 66.04193 |
| 3 | 61.04034 | 62.03736 | 62.04367 | 66.04197 |
| 4 | 61.04030 | 62.03727 | 62.04359 | 66.04191 |
| 5 | 61.04032 | 62.03729 | 62.04362 | 66.04194 |
| 6 | 61.04031 | 62.03729 | 62.0436 | 66.04193 |
| 7 | 61.04032 | 62.03736 | 62.04364 | 66.04195 |
| 8 | 61.04030 | 62.03735 | 62.04363 | 66.04194 |
| 9 | 61.04033 | 62.03736 | 62.04364 | 66.04196 |
| 10 | 61.04017 | 62.03717 | 62.04348 | 66.04179 |
| 11 | 61.04017 | 62.03717 | 62.04347 | 66.04178 |
| 12 | 61.04014 | 62.03715 | 62.04345 | 66.04176 |
| 13 | 61.04033 | 62.03729 | 62.04361 | 66.04194 |
| 14 | 61.04032 | 62.03729 | 62.04361 | 66.04194 |
| 15 | 61.04032 | 62.03729 | 62.04361 | 66.04194 |
| 16 | 61.04040 | 62.03738 | 62.0437 | 66.04202 |
| 17 | 61.04039 | 62.03737 | 62.0437 | 66.04202 |
| 18 | 61.04040 | 62.03738 | 62.04371 | 66.04203 |
| 19 | 61.04022 | 62.03716 | 62.04350 | 66.04182 |
| 20 | 61.04022 | 62.03717 | 62.04350 | 66.04182 |
| 21 | 61.04021 | 62.03716 | 62.0435 | 66.0418 |
| 22 | 61.04020 | 62.03718 | 62.0435 | 66.04181 |
| 23 | 61.04021 | 62.03719 | 62.04352 | 66.04181 |
| 24 | 61.04021 | 62.03719 | 62.04351 | 66.04181 |
| Mean | 61.04028 | 62.03727 | 62.04358 | 66.04189 |
| Stdev | 7.56E-05 | 8.53E-05 | 8.00E-05 | 8.39E-05 |
| Stdev (ppm) | 1.23951 | 1.37626 | 1.29027 | 1.27182 |
| Theoretical Mass | 61.04019 | 62.03722 | 62.043543 | 66.041857 |
| **Accuracy (ppm)** | **1.49** | **0.83** | **0.67** | **0.63** |
| **RMS (ppm)** | **0.06** | **0.08** | **0.07** | **0.07** |

**Supplementary Table 4:** Selectivity for isotopically labeled amino acids as measured in m/z for the respective protonated species.  

| **Injection** | **[[D**_**5**_**]Phenylalanine+H]**^**+**^   **(*****m/z*****)** | **[Citrulline+H]**^**+**^   **(*****m/z*****)** | **[[**^**15**^**N]Citrulline+H]**^**+**^   **(*****m/z*****)** | **[[**^**13**^**C]Citrulline+H]**^**+**^   **(*****m/z*****)** |
| --- | --- | --- | --- | --- |
| 1 | 341.16537 | 346.15066 | 347.14794 | 347.1539 |
| 2 | 341.1654 | 346.15072 | 347.14778 | 347.15395 |
| 3 | 341.1654 | 346.15076 | 347.14784 | 347.15396 |
| 4 | 341.16549 | 346.15068 | 347.14804 | 347.15392 |
| 5 | 341.1654 | 346.15069 | 347.14785 | 347.15397 |
| 6 | 341.16559 | 346.1508 | 347.14794 | 347.15412 |
| 7 | 341.16536 | 346.15066 | 347.14789 | 347.15391 |
| 8 | 341.16536 | 346.1507 | 347.14775 | 347.15397 |
| 9 | 341.16539 | 346.15077 | 347.14786 | 347.15405 |
| 10 | 341.16463 | 346.14986 | 347.14731 | 347.15318 |
| 11 | 341.16444 | 346.14986 | 347.14699 | 347.15312 |
| 12 | 341.16466 | 346.14994 | 347.14715 | 347.15321 |
| 13 | 341.16518 | 346.15054 | 347.14794 | 347.1537 |
| 14 | 341.16518 | 346.1506 | 347.15379 | 347.14798 |
| 15 | 341.16532 | 346.15064 | 347.14800 | 347.1538 |
| 16 | 341.16564 | 346.15091 | 347.14812 | 347.15416 |
| 17 | 341.16559 | 346.1509 | 347.14803 | 347.1542 |
| 18 | 341.1655 | 346.15092 | 347.14801 | 347.15426 |
| 19 | 341.16532 | 346.15065 | 347.14795 | 347.15387 |
| 20 | 341.16521 | 346.15053 | 347.14777 | 347.15382 |
| 21 | 341.16522 | 346.1506 | 347.14777 | 347.15386 |
| 22 | 341.16458 | 346.14995 | 347.1473 | 347.15314 |
| 23 | 341.16459 | 346.1499 | 347.14719 | 347.15305 |
| 24 | 341.16472 | 346.15001 | 347.14722 | 347.15321 |
| Mean | 341.16519 | 346.15051 | 347.14798 | 347.15351 |
| Stdev | 0.00037 | 0.00036 | 0.00128 | 0.00124 |
| Stdev (ppm) | 1.08 | 1.05 | 3.69 | 3.57 |
| Theoretical Mass | 341.16565 | 346.1515 | 347.1485 | 347.1548 |
| **Accuracy (ppm)** | **-1.36** | **-2.86** | **-1.50** | **-3.71** |
| **RMS (ppm)** | **0.05** | **0.05** | **0.57** | **0.53** |

**Supplementary Table 5:** Intra-experiment variability in plasma.

| **Metabolite** | **Time (min)** | **Average [**^**15**^**N]  isotopic ratio(%)** | **Standard deviation (%)** | **CV (%), n=18** |
| --- | --- | --- | --- | --- |
| **[**^**15**^**N]Urea** | 0 | 0.7826 | 0.0128 | **1.63** |
|  | 5 | 0.9687 | 0.0159 | **1.64** |
|  | 8 | 1.1751 | 0.0187 | **1.59** |
|  | 30 | 1.3882 | 0.0179 | **1.29** |
| **[**^**15**^**N]Arginine** | 0 | 1.7529 | 0.0585 | **3.34** |
|  | 5 | 2.0665 | 0.1279 | **6.19** |
|  | 8 | 2.7192 | 0.1137 | **4.18** |
|  | 30 | 3.8804 | 0.1170 | **3.02** |
| **[**^**15**^**N]Citrulline** | 0 | 1.3997 | 0.0836 | **5.97** |
|  | 5 | 4.5465 | 0.1679 | **3.69** |
|  | 8 | 9.8847 | 0.1794 | **1.82** |
|  | 30 | 18.4575 | 0.3721 | **2.02** |
| **[**^**15**^**N]Glutamine** | 0 | 1.1278 | 0.0464 | **4.12** |
|  | 5 | 1.2421 | 0.0519 | **4.17** |
|  | 8 | 1.5772 | 0.0522 | **3.31** |
|  | 30 | 2.8485 | 0.0513 | **1.80** |
| **[**^**15**^**N]Glycine** | 0 | 0.9579 | 0.0276 | **2.88** |
|  | 5 | 0.9719 | 0.0244 | **2.52** |
|  | 8 | 0.9931 | 0.0205 | **2.06** |
|  | 30 | 1.4914 | 0.0267 | **1.79** |
| **[**^**15**^**N]Glutamate** | 0 | 0.7997 | 0.0700 | **8.75** |
|  | 5 | 1.3550 | 0.0550 | **4.06** |
|  | 8 | 2.9755 | 0.0744 | **2.50** |
|  | 30 | 2.9703 | 0.0526 | **1.77** |

**Supplementary Table 6:** Intra-experiment variation in dried blood spots (DBS).

| **Metabolite** | **Time (min)** | **Average [**^**15**^**N]  isotopic ratio(%)** | **Standard deviation (%)** | **CV (%), n=18** |
| --- | --- | --- | --- | --- |
| **[**^**15**^**N]Urea** | 0 | 0.7286 | 0.0198 | **2.72** |
|  | 5 | 0.9007 | 0.0268 | **2.98** |
|  | 8 | 1.1105 | 0.0187 | **1.69** |
|  | 30 | 1.2983 | 0.0295 | **2.27** |
| **[**^**15**^**N]Arginine** | 0 | 1.2687 | 0.3003 | **23.67** |
|  | 5 | 1.4821 | 0.2202 | **14.86** |
|  | 8 | 1.7232 | 0.3078 | **17.86** |
|  | 30 | 2.2133 | 0.2984 | **13.48** |
| **[**^**15**^**N]Citrulline** | 0 | 1.3155 | 0.1443 | **10.97** |
|  | 5 | 3.1285 | 0.1425 | **4.56** |
|  | 8 | 6.1967 | 0.2475 | **3.99** |
|  | 30 | 11.0944 | 0.2681 | **2.42** |
| **[**^**15**^**N]Glutamine** | 0 | 1.0974 | 0.0481 | **4.38** |
|  | 5 | 1.1895 | 0.0557 | **4.68** |
|  | 8 | 1.3767 | 0.0448 | **3.25** |
|  | 30 | 2.1734 | 0.0577 | **2.65** |
| **[**^**15**^**N]Glycine** | 0 | 0.9660 | 0.0358 | **3.70** |
|  | 5 | 0.9496 | 0.0422 | **4.44** |
|  | 8 | 0.9717 | 0.0348 | **3.59** |
|  | 30 | 1.2081 | 0.0378 | **3.13** |
| **[**^**15**^**N]Glutamate** | 0 | 0.7788 | 0.0362 | **4.65** |
|  | 5 | 0.9272 | 0.0692 | **7.46** |
|  | 8 | 1.1929 | 0.0568 | **4.76** |
|  | 30 | 1.1728 | 0.0564 | **4.81** |

**Supplementary Table 7:** Inter-experiment variability in plasma.

| **Metabolite** | **Time (min)** | **Average [**^**15**^**N]  isotopic ratio (%)** | **Standard deviation (%)** | **CV (%), n=18** |
| --- | --- | --- | --- | --- |
| **[**^**15**^**N]Urea** | 0 | 0.7629 | 0.0268 | **3.51** |
|  | 5 | 0.9530 | 0.0181 | **1.90** |
|  | 8 | 1.1542 | 0.0351 | **3.04** |
|  | 30 | 1.3834 | 0.0299 | **2.16** |
| **[**^**15**^**N]Arginine** | 0 | 1.6713 | 0.1283 | **7.68** |
|  | 5 | 2.0200 | 0.1700 | **8.42** |
|  | 8 | 2.6848 | 0.1198 | **4.46** |
|  | 30 | 3.8253 | 0.1344 | **3.51** |
| **[**^**15**^**N]Citrulline** | 0 | 1.4619 | 0.1001 | **6.85** |
|  | 5 | 4.6607 | 0.1651 | **3.54** |
|  | 8 | 10.0597 | 0.1981 | **1.97** |
|  | 30 | 18.5189 | 0.2836 | **1.53** |
| **[**^**15**^**N]Glutamine** | 0 | 1.1156 | 0.0992 | **8.89** |
|  | 5 | 1.2753 | 0.0612 | **4.80** |
|  | 8 | 1.5594 | 0.0513 | **3.29** |
|  | 30 | 2.8584 | 0.0683 | **2.39** |
| **[**^**15**^**N]Glycine** | 0 | 0.9497 | 0.0312 | **3.29** |
|  | 5 | 0.9718 | 0.0266 | **2.73** |
|  | 8 | 0.9866 | 0.0281 | **2.84** |
|  | 30 | 1.5053 | 0.0355 | **2.36** |
| **[**^**15**^**N]Glutamate** | 0 | 0.7829 | 0.0594 | **7.59** |
|  | 5 | 1.4984 | 0.1089 | **7.27** |
|  | 8 | 3.1115 | 0.1596 | **5.13** |
|  | 30 | 3.0793 | 0.1031 | **3.35** |

**Supplementary Table 8:** Inter-experiment variability in dried blood spots (DBS).

| **Metabolite** | **Time (min)** | **Average [**^**15**^**N]  isotopic ratio(%)** | **Standard deviation** | **CV (%), n=18** |
| --- | --- | --- | --- | --- |
| **[**^**15**^**N]Urea** | 0 | 0.7288 | 0.0224 | **3.07*** |
|  | 5 | 0.9066 | 0.0378 | **4.17*** |
|  | 8 | 1.1051 | 0.0273 | **2.47*** |
|  | 30 | 1.3075 | 0.0435 | **3.33*** |
| **[**^**15**^**N]Arginine** | 0 | 1.4458 | 0.2147 | **14.85** |
|  | 5 | 1.6086 | 0.1462 | **9.09** |
|  | 8 | 1.9136 | 0.1377 | **7.20** |
|  | 30 | 2.4129 | 0.2645 | **10.96** |
| **[**^**15**^**N]Citrulline** | 0 | 1.3589 | 0.1207 | **8.88** |
|  | 5 | 3.2278 | 0.1351 | **4.19** |
|  | 8 | 6.3587 | 0.1989 | **3.13** |
|  | 30 | 11.2301 | 0.2864 | **2.55** |
| **[**^**15**^**N]Glutamine** | 0 | 1.1089 | 0.0364 | **3.28** |
|  | 5 | 1.2043 | 0.0415 | **3.45** |
|  | 8 | 1.4028 | 0.0453 | **3.23** |
|  | 30 | 2.1991 | 0.0816 | **3.71** |
| **[**^**15**^**N]Glycine** | 0 | 0.9469 | 0.0317 | **3.34** |
|  | 5 | 0.9650 | 0.0248 | **2.57** |
|  | 8 | 0.9823 | 0.0282 | **2.87** |
|  | 30 | 1.1929 | 0.0277 | **2.33** |
| **[**^**15**^**N]Glutamate** | 0 | 0.7531 | 0.0446 | **5.92** |
|  | 5 | 0.9357 | 0.0868 | **9.28** |
|  | 8 | 1.2012 | 0.0650 | **5.41** |
|  | 30 | 1.2054 | 0.0389 | **3.23** |

*Urea measurements for DBS only contain data from four different replicates (n=12).

**Supplementary Table 9:** LLOQD for [^15^N] isotopic enrichment in urea cycle metabolites.

| **Matrix** | **Metabolite** | **[**^**15**^**N] Isotopic ratio (%)** | **Standard deviation (%)** | **LLOQD (%)** |
| --- | --- | --- | --- | --- |
| **Plasma** | **[**^**15**^**N]Urea** | 0.7826 | 0.0128 | **0.0384** |
|  | **[**^**15**^**N]Arginine** | 1.7529 | 0.0585 | **0.1755** |
|  | **[**^**15**^**N]Citrulline** | 1.3997 | 0.0836 | **0.2508** |
|  | **[**^**15**^**N]Glutamine** | 1.1278 | 0.0464 | **0.1392** |
|  | **[**^**15**^**N]Glycine** | 0.9579 | 0.0276 | **0.0828** |
|  | **[**^**15**^**N]Glutamate** | 0.7997 | 0.0700 | **0.2100** |
| **DBS** | **[**^**15**^**N]Urea** | 0.7286 | 0.0198 | **0.0594** |
|  | **[**^**15**^**N]Arginine** | 1.2687 | 0.3003 | **0.9009** |
|  | **[**^**15**^**N]Citrulline** | 1.3155 | 0.1443 | **0.4329** |
|  | **[**^**15**^**N]Glutamine** | 1.0974 | 0.0481 | **0.1443** |
|  | **[**^**15**^**N]Glycine** | 0.9660 | 0.0358 | **0.1074** |
|  | **[15N]Glutamate** | 0.7788 | 0.0362 | **0.1086** |

**Supplementary Table 10:** Average relative difference in [^15^N] isotope ratio, calculated as the average difference from all time-points for each metabolite per test subject as well as cumulative over all subjects.

|  | **Average difference (%)** |  |  |  |
| --- | --- | --- | --- | --- |
|  | **P1** | **P2** | **P3** | **Cumulative** |
| **Urea** | -2.83 | 2.19 | -4.96 | **-1.87** |
| **Citrulline** | 4.42 | -2.04 | -2.90 | **-0.17** |
| **Arginine** | -6.21 | -0.54 | -11.67 | **-6.14** |
| **Glutamine** | 0.02 | 1.48 | -2.19 | **-0.23** |
| **Glycine** | 2.28 | -0.79 | -0.30 | **0.40** |
| **Glutamate** | -12.08 | -7.44 | -16.65 | **-12.06** |

**Supplementary Table 11:** Average relative difference in [^15^N] isotope ratio due to delayed sample processing.

|  | **Average difference (%)** |  |
| --- | --- | --- |
|  | **15 minutes** | **30 minutes** |
| **[**^**15**^**N]Urea** | 0.13 | 1.27 |
| **[**^**15**^**N]Citrulline** | 1.71 | 3.19 |
| **[**^**15**^**N]Arginine** | -3.86 | 3.13 |
| **[**^**15**^**N]Glutamine** | 0.19 | 1.15 |
| **[**^**15**^**N]Glycine** | 0.03 | -0.41 |
| **[**^**15**^**N]Glutamate** | 3.96 | 3.86 |

**Supplementary Table 12:** Unlabeled and isotopically labeled metabolites including their protonated mass formula and exact mass

| **Metabolite** | **Protonated Mass Formula** | **Protonated Exact mass (g/mol)** |
| --- | --- | --- |
| Urea | CH_5_N_2_O | 61.04019 |
| [^13^C]urea | ^13^CH_5_N_2_O | 62.04354 |
| [^15^N]-urea | CH_5_^15^NNO | 62.03722 |
| [^15^N_2_]-urea | CH_5_^15^N_2_O | 63.03426 |
| IS-Urea | ^13^CH_5_^15^N_2_^18^O | 66.04186 |
| **Derivatized amino acids** |  |  |
| Glutamine | C_15_H_17_N_4_O_4_ | 317.1249 |
| [^15^N]Glutamine | C_15_H_17_^15^NN_3_O_4_ | 318.1220 |
| [^13^C]Glutamine | ^13^CC_14_H_17_N_2_O_4_ | 318.1283 |
| Citrulline | C_16_H_20_N_5_O_4_ | 346.1515 |
| [^15^N]Citrulline | C_16_H_20_^15^NN_4_O_4_ | 347.1485 |
| [^13^C]Citrulline | ^13^CC_15_H_20_N_5_O_4_ | 347.1548 |
| Arginine | C_16_H_21_N_6_O_3_ | 345.1675 |
| [^15^N]Arginine | C_16_H_21_^15^NN_5_O_3_ | 346.1645 |
| [^13^C]Arginine | ^13^CC_15_H_21_N_6_O_3_ | 346.1708 |
| Ornithine | C_5_H_13_N_2_O_2_ | 133.0977 |
| [^15^N]Ornithine | C_5_H_13_^15^NNO_2_ | 134.0947 |
| [^13^C]Ornithine | ^13^CC_4_H_13_N_2_O_2_ | 134.1011 |
| Argininosuccinic acid | C_20_H_25_N_6_O_7_ | 461.1784 |
| [^15^N]Argininosuccinic acid | C_20_H_25_^15^NN_5_O_7_ | 462.1755 |
| [^13^C]Argininosuccinic acid | ^13^CC_19_H_25_N_6_O_7_ | 462.1818 |
| Glutamate | C_15_H_16_N_3_O_5_ | 318.1090 |
| [^15^N]Glutamate | C_15_H_16_^15^NN_2_O_5_ | 319.1060 |
| [^13^C]Glutamate | ^13^CC_14_H_16_N_3_O_5_ | 319.1123 |
| Glycine | C_12_H_12_N_3_O_3_ | 246.0878 |
| [^15^N]Glycine | C_12_H_12_^15^NN_2_O_3_ | 247.0849 |
| [^13^C]Glycine | ^13^CC_11_H_12_N_3_O_3_ | 247.0912 |
| [D_5_]Phenylalanine | C_19_D_5_H_13_N_3_O_3_ | 341.1662 |

**Supplementary Figures**

**Supplementary Figure 1:** Body mass index (BMI) and tracer recovery

**Supplementary Fig 1**: **A**. Body mass index (BMI) and tracer recovery; **B**. tracer recovery per disease group. Half colored symbols represent treated patients. BMI was classified according to CDC (Center of Disease Control). Children less than 2 years-old does not have a classification for BMI. 
**Supplementary Figure 2:** [^15^N]glycine enrichment for citrin deficiency patients. 

**Supplementary Fig. 2**: Average ± standard error of the mean of [^15^N]glycine enrichment for citrin deficiency (CTND) patients (n=6) and controls (CTRL, in black).

**Supplementary Figure 3:** Separation of [^15^N] and [^13^C] labeled urea and citrulline

**Supplementary** **Figure 3:** Separation of [^15^N] and [^13^C] labeled urea and citrulline. For urea, the full scan range was set to 50.0 to 100.1 m/z and spectra were recorded in the profile spectrum data at a scan rate of 0.1 s/scan (maximum injection time: 0.1 s) and at a resolution of 70,000 (FWHM). For citrulline and other amino acids analysis, the full scan range was set from 100.0 to 500.1 m/z and spectra were recorded in profile spectrum mode at a scan rate of 0.1 s/scan (maximum injection time: 0.1 s) and at a resolution of 140,000 (FWHM). 

**Supplementary** **Figure 4:** Linear isotopic enrichment

**Supplementary** **Figure 4:** Linear isotopic enrichment was obtained using human hepatocyte cell cultures derived from induced pluripotent stem cells (iPSCs) that were incubated with three different concentration (0 mM, 1 mM and 2 mM) of the [^15^N]H_4_Cl tracer for 24 h in triplicates per time concentration, since no standard of [^15^N]urea was commercially available. 

**Supplementary** **Figure 5:** Sample stability after one year at -80°C from three different subjects 

**Supplementary** **Figure 5:** Sample stability after one year at -80°C from three different subjects. Samples indexed with “_1” were immediately measured and the ones with “_2” were measured 1 year after collection. 

**Supplementary** **Figure 6:** Effects of delayed sample processing on the [^15^N] isotope ratio of ureagenesis metabolites and related amino acids

**Supplementary** **Figure 6:** Effects of delayed sample processing on the [^15^N] isotope ratio of ureagenesis metabolites and related amino acids.

**Supplementary** **Figure 7:** Comparison of plasma and dried blood spot samples.

**Supplementary Figure 7:** **A.** Plot of samples from patients and controls that were measured in plasma and DBS (n=49). **B.** Bland-Altman plot of the %Difference vs average. The values above and below the dotted lines (95% limits of agreement) are from patients with RUF close to zero.

**Supplementary Figure 8:** [^15^N] enrichment curves for urea under various conditions

**Legend to Supplementary Figure 3:** Graphical analysis of [^15^N] enrichment curves for urea for a patient with urea cycle defect (**A**). Examples of normalization to tracer amount, where the subject received the double amount of tracer (**B**), and endogenous metabolite concentration, where the subject’s plasma urea concentration was 4 mM compared to 5.5 mM in controls (**C**). Example of patient (**D**) with highly variable endogenous metabolite concentration during the test, in which the results for this specific metabolite cannot be interpreted quantitatively. Controls are shown as average ± standard deviation.
